# Consecutive isocyanide-based multicomponent reactions: synthesis of cyclic pentadepsipeptoids

**DOI:** 10.3762/bjoc.10.101

**Published:** 2014-05-05

**Authors:** Angélica de Fátima S Barreto, Otilie E Vercillo, Ludger A Wessjohann, Carlos Kleber Z Andrade

**Affiliations:** 1 Laboratório de Química Metodológica e Orgânica Sintética, Instituto de Química, Universidade de Brasília, CP 4478, 70910-970 Brasília-DF, Brazil; 2 Faculdade UnB Planaltina, Área Universitária Nº 1, Vila Nossa Senhora de Fátima, Planaltina, 73300-000, Brasília, DF, Brazil; 3 Department of Bioorganic Chemistry, Leibniz Institute of Plant Biochemistry, Weinberg 3, D-06120 Halle (Saale), Germany

**Keywords:** depsipeptoids, multicomponent reactions, Passerini reaction, sansalvamide A, Ugi reaction

## Abstract

The synthesis of six cyclic depsipeptoids inspired by the natural depsipeptide sansalvamide A is described. An efficient and fast synthetic strategy was developed using a combination of consecutive isocyanide-based multicomponent reactions (Ugi and Passerini reactions). This methodology can be used to access a variety of cyclic oligodepsipeptoids.

## Introduction

Peptoids are an interesting class of non-natural compounds that have recently received much attention due to their wide range of biological activities, which makes them attractive candidates for drug discovery [1–7]. This family of oligomers comprising poly-*N*-substituted glycines mimics the primary natural structure of peptides and exhibits greater proteolytic stability and increased cellular permeabilities in comparison to peptides [5–7]. A powerful synthetic tool for the preparation of a peptoid backbone is the Ugi four-component reaction (U-4CR) [8–14]. It has been demonstrated that the combination of multicomponent reactions with the use of microwave irradiation is able to efficiently produce complex molecules with a reduced number of steps and short reaction times [15–18].

**Figure 1:**
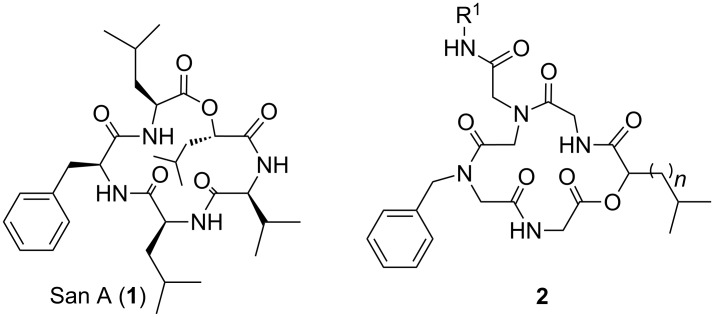
Sansalvamide A (**1**) and its depsipeptoid analogues (**2**).

Depsipeptides are polymeric natural compounds, analogues of peptides, being formed by amino acids and hydroxy acids linked together by amide and ester bonds. These natural products show promising biological activities, especially regarding their therapeutic potential in cancer treatment [19]. An example of a cyclic depsipeptide is sansalvamide A (San A, Figure 1) [20–29], which was isolated from a marine fungus (*Fusarium spp.*) [20] and exhibits antitumor activity against multiple cancer cell lines. It is cytotoxic against colon (HCT-116) [20,23,25–26], pancreatic (S2-013 and AsPC-1) [22,28–29], prostate (PC-3), breast (MDA-MB231) and melanoma cancers (WM-115) [24]. It has been reported that substitution of an ester group by an amide in the structure of a peptide provides an efficient way to evaluate the role of protein hydrogen bonding [30–34]. Recent works have discovered that some analogues of San A inhibit Hsp 90, a key protein that enables many proteins involved in tumor progression [35–39].

**Figure 2:**
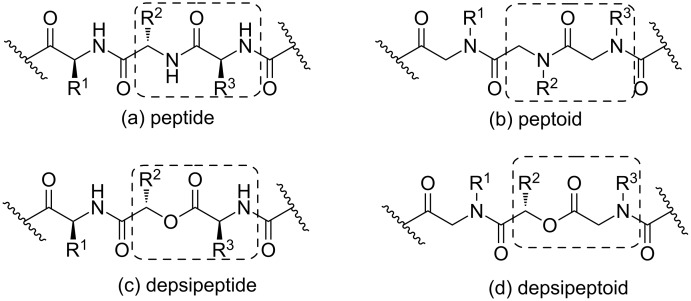
Generic structures of (a) peptide, (b) peptoid, (c) depsipeptide and (d) depsipeptoid.

The Passerini three-component reaction (P-3CR) allows an easy access to depsipeptides using a convergent approach. It has become a powerful tool in combinatorial synthesis [40–43] and can be used strategically for the synthesis of depsipeptoids. By analogy to peptides and peptoids, a depsipeptoid would be a peptoid bearing an ester group instead of an amide group. Differences between peptide, peptoid, depsipeptide and depsipeptoid structures are outlined in Figure 2.

## Results and Discussion

**Figure 3:**
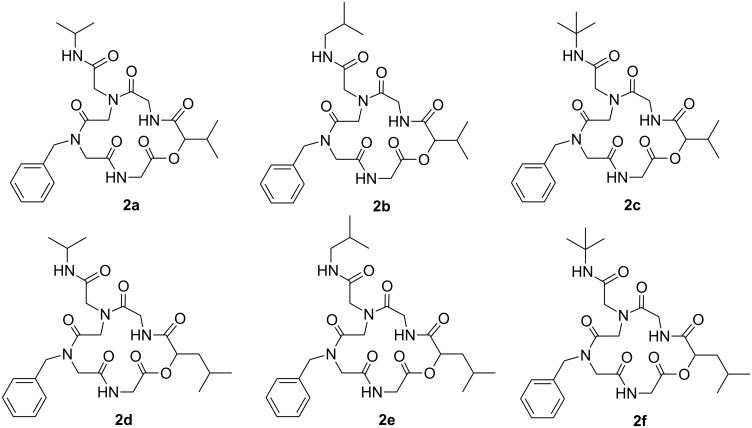
Structures of six pentadepsipeptoid analogues of San A.

In continuing our research on the synthesis of peptoids with potential pharmacological activity [12,17–18,44–45] and using a fast and efficient microwave-assisted synthesis of peptoids [15,17–18], we decided to carry out the synthesis of depsipeptoid analogues of San A based on a strategy developed in our groups for the synthesis of cyclic RGD pentapeptoids [44]. This strategy was adapted by a combination of microwave-assisted Ugi and Passerini reactions. It is also important to highlight that the synthesis of cyclic depsipeptoids had not been explored yet. In this paper, we describe the synthesis of six pentadepsipeptoid analogues of San A (Figure 3).

The synthetic route for the synthesis of cyclic pentadepsipeptoids via consecutive Ugi reactions allows only three side chains connected to three nitrogen atoms. The pentapeptide of San A has in its structure five side chains attached to the α carbon atoms: one isopropyl, one benzyl and three isobutyl groups. To generate the pentapeptoid analogues of San A, the side chain groups isobutyl, isopropyl and benzyl linked to the α carbon atom present in the peptide were moved to the nitrogen atoms. It was decided to keep at least one benzyl group in the structure of the peptoids and vary the isopropyl and isobutyl groups, thus maintaining a greater similarity with the structure of the San A depsipeptide.

**Scheme 1:**
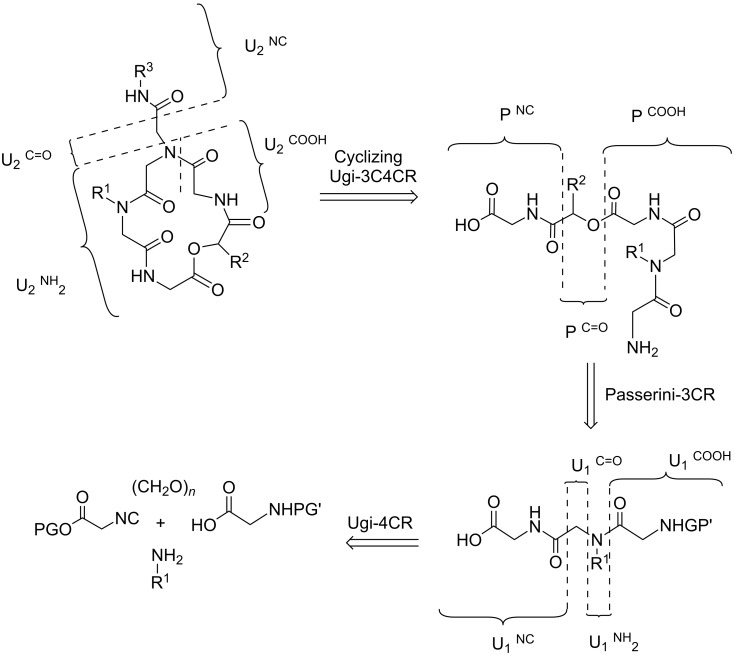
Retrosynthetic analysis of the cyclic depsipeptoids.

The retrosynthetic analysis of the depsipeptoids (Scheme 1) shows that the proposed compounds can be achieved using a strategy based on: (a) formation of a peptoid via Ugi reaction; (b) ester hydrolysis; (c) formation of an acyclic depsipeptoid scaffold through a Passerini reaction; (d) deprotection of the amine/acid groups and (e) a macrocyclization step via an intramolecular Ugi reaction.

**Scheme 2:**
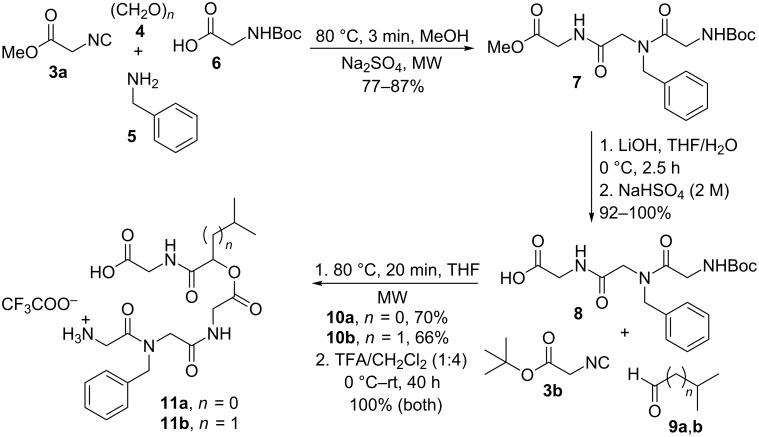
Synthesis of acyclic depsipeptoids **11a**,**b**.

The general strategy for the synthesis of cyclic pentadepsipeptoids is depicted in Scheme 2. The synthesis of analogues **2** was initiated by an Ugi 4-component reaction (U-4CR) using methyl isocyanoacetate (**3a**), paraformaldehyde (**4**)**,** benzylamine (**5**) and *N*-Boc-glycine (**6**) in MeOH (Scheme 2) in a microwave (MW) reactor (3 min at 80 °C) to provide the peptoid **7** in 77–87% yield. Peptoid **7** was subjected to hydrolysis in the presence of LiOH (THF/H_2_O, 0 °C, 2.5 h) followed by treatment with 2 M NaHSO_4_ providing the corresponding acid **8** in 92–100% yield. Acid **8** was then employed in a Passerini reaction with isobutyraldehyde (**9a**) or isovaleraldehyde (**9b**) and *tert-*butyl isocyanoacetate (**3b**), in a MW reactor (20 min at 80 °C) in THF, affording the acyclic depsipeptoids **10a** and **10b** in 70% and 66% yield, respectively. Removal of the *Boc* protecting group and ester hydrolysis were achieved after treatment of the acyclic depsipeptoids **10a**,**b** with TFA in CH_2_Cl_2_, giving the corresponding amino acids **11a**,**b** as TFA salt in quantitative yields.

**Scheme 3:**
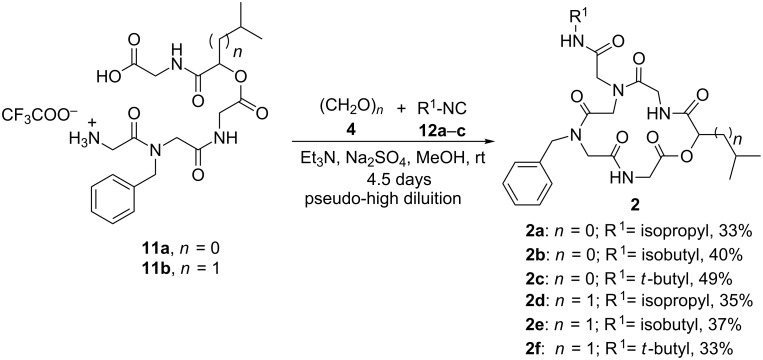
Synthesis of macrocycles **2a-f**.

In the last step, the depsipeptoid amino acid salts **11a**,**b** were subjected to an Ugi three-component four-center reaction (U-3C4CR) (Scheme 3). Compounds **11a**,**b** were added under pseudo-high dilution conditions (addition rate: 0.6 mL/h; addition time: 83 h; concentration: 0.80 mmol/L) to a suspension of paraformaldehyde **4**, triethylamine, sodium sulfate and isopropyl/isobutyl/*tert*-butyl isocyanide **12a–c** in methanol to yield the target cyclic pentadepsipeptoids **2a–f** after purification by column chromatography (yields ranged from 33–49% depending on the substrate). The structures of the final products obtained are shown in Figure 3.

## Conclusion

In summary, the approach developed herein allows the synthesis of a wide range of cyclic depsipeptoids. Different structures can be obtained by changes in the amine component in the first Ugi reaction, in the carbonyl component in the Passerini reaction or in the isocyanide and carbonyl components in the macrocyclization step. The general route and procedure developed allows an easy access to complex molecules with a significantly reduced number of steps in short reaction times, and high yields in most of the steps. The strategic combination of two isocyanide-based multicomponent reactions and microwave irradiation makes this a very useful and attractive protocol. The obtained depsipeptoids will be tested for different biological activities.

## Supporting Information

File 1 General procedures, NMR and mass spectra of all compounds.
