# Supplementary material for: Consecutive isocyanide-based multicomponent reactions: synthesis of cyclic pentadepsipeptoids
Source: Beilstein J Org Chem. 2014 May 5;10:1017–22. doi: 10.3762/bjoc.10.101 (PMC4077530; doi:10.3762/bjoc.10.101)
Supplement: File 1 — General procedures, NMR and mass spectra of all compounds. [file Beilstein_J_Org_Chem-10-1017-s001.pdf]

Supporting Information

for

Consecutive isocyanide-based multicomponent reactions: synthesis of cyclic pentadepsipeptoids

Angélica de Fátima S. Barreto[1], Otilie E. Vercillo[2], Ludger A. Wessjohann[3] and Carlos Kleber Z. Andrade[1]*

Address: [1]Laboratório de Química Metodológica e Orgânica Sintética, Instituto de Química, Universidade de Brasília, CP 4478, 70910-970 Brasília-DF, Brazil; [2] Faculdade UnB Planaltina, Área Universitária Nº 1, Vila Nossa Senhora de Fátima, Planaltina, 73300-000, Brasília, DF, Brazil and [3]Department of Bioorganic Chemistry, Leibniz Institute of Plant Biochemistry, Weinberg 3, D-06120 Halle (Saale), Germany.

Email: Carlos Kleber Z. Andrade - ckleber@unb.br

* Corresponding author

General procedures, NMR and mass spectra of all compounds

List of contents

General information S2

Experimental section S2

General procedure for the Passerini reaction S3

General procedure for the deprotection S5

General procedure for macrocyclization via intramolecular Ugi reactions S6

Spectra of selected compounds S11–S44

General information

NMR spectra were recorded on a Varian Mercury Plus 300 spectrometer at 300 MHz for ^{1}H and 75.46 MHz for ^{13}C in the presence of TMS as internal standard. TLC plates were revealed by treatment with a 10% solution of phosphomolybdic acid in ethanol, followed by heating. High resolution ESI mass spectra were obtained on a Micro TOF- Bruker Daltonics instrument and IR spectra were recorded on a Varian 640-IR spectrometer. Reactions were performed on a CEM Co., Discover microwave reactor using sealed vessels, dynamic program, temperature detection by internal fiber optic probe, simultaneous cooling and media stirring. Melting points were recorded on a Logen Scientific-LS melting point apparatus and are uncorrected. Commercially available reagents and solvents were analytical grade or were purified by standard procedures prior to use. Compounds were analyzed by IR, ^{1}H NMR, ^{13}C NMR and high resolution ESI mass spectra giving data consistent with the proposed structures.

Experimental section

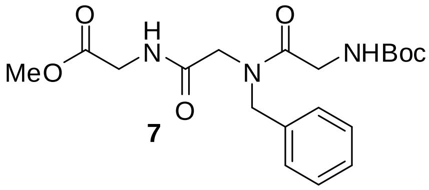

7

Preparation of peptoid 7 via Ugi reaction [1]: A sealed 10 mL glass tube containing a mixture of benzylamine (**5**, 0.321 g, 3.00 mmol), methanol (1.5 mL), anhydrous sodium sulfate (0.90 g), paraformaldehyde (**4**, 0.090 g, 3.00 mmol), *Boc*-glycine (**6**, 0.263 g, 1.50 mmol) and methyl isocyanoacetate (**3a**, 0.136 mL, 1.50 mmol) was introduced in the cavity of a microwave reactor (CEM Co., Discover) and irradiated at 80 ºC for 3 min (ramp time: 93 s) under magnetic stirring. The residue was filtered, concentrated in vacuum and purified by column chromatography (CH_2Cl_2→ 1% MeOH/CH_2Cl_2) to yield peptoid **7** (0.470 g, 1.20 mmol, 80%) as a yellow viscous oil. R_f (3% MeOH/CH_2Cl_2)= 0.25. IR 3426, 2978, 1747, 1654, 1219, 1168, 736, 697 cm^{-1}. ^{1}H NMR (300 MHz, $CDCl_3$): δ 7.38-7.18 (m, 5H), 7.11 (br s, 1H), 5.67 and 5.61 (2br s, 1H), 4.67 and 4.63 (2br s, 2H), 4.08-3.93 (m, 6H), 3.72 (s, 3H), 1.42 (s, 9H). ^{13}C NMR (75.46 MHz, $CDCl_3$): δ 170.1, 168.6, 168.0, 155.8, 134.8, 129.0, 128.6, 128.3, 128.0, 126.7, 79.7, 52.2, 51.3, 49.2, 42.1, 40.8, 28.2. HRMS (ESI) *m/z*: calc. for $[M+Na]^+$ $C_{19}H_{27}N_3O_6Na$: 416.1798; found: 416.1805.

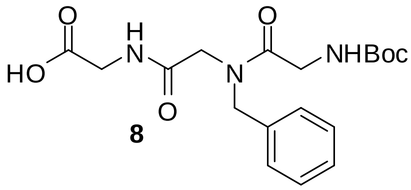

8

Hydrolysis of 7: To a solution of the peptoid **7** (0.470 g, 1.20 mmol) in THF/H_2O (2:1, 42.0 mL) was added LiOH (0.143 g, 5.97 mmol) at 0 ºC. The reaction was stirred for 2.5 h at 0 °C. After completion, the reaction mixture was acidified with a 2 M solution of $NaHSO_4$ to pH 2 and extracted twice with ethyl acetate. The organic phase was dried with sodium sulfate, filtered and concentrated to yield acid **8** (0.417 g, 1.10 mmol) in 92% yield as white solid, which was used without further purification. M.p. (from ethyl acetate) = 147-149 ºC. IR 3434, 3313, 3278, 2991, 2936, 1765, 1670, 1647, 1551, 1384, 1310, 1231, 1174, 1033, 748 cm^{-1}. ^{1}H NMR (300 MHz, CD_3OD, presence of rotamers): δ 7.40-7.25 (m, 5H), 4.66 and 4.63 (2s, 2H), 4.10-4.03 (m, 3H), 3.97 and 3.92 (2s, 3H), 1.45 and 1.43 (2s, 9H). ^{13}C NMR (75.46 MHz, CD_3OD, presence of rotamers): δ 172.9, 172.5, 171.4, 158.5, 137.8, 130.1, 129.7, 129.3, 129.0, 128.2, 80.6, 52.2, 51.1, 43.3, 41.8, 28.7. HRMS (ESI) *m/z*: calc. for $[M+Na]^+$ $C_{18}H_{25}N_3O_6Na$: 402.1641; found: 402.1628.

General procedure for the Passerini reaction [2]:

A sealed 10 mL glass tube containing a mixture of aldehyde (1.4 equiv), carboxylic acid (1.0 equiv) and isocyanide (1.4 equiv) in THF was introduced in the cavity of a microwave reactor (CEM Co., Discover). The glass tube was irradiated at 80 °C for 20 min (ramp time specified below for each compound) under magnetic stirring. After completion, the reaction mixture was diluted in CH_2Cl_2, concentrated in vacuum and purified by column chromatography to yield the corresponding acyclic depsipeptoids **10a** and **10b**.

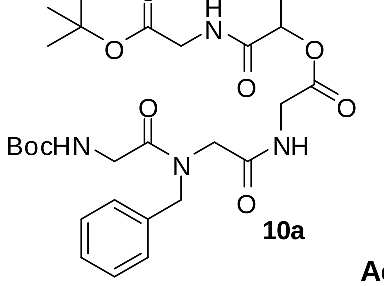

10a

Acyclic depsipeptoid 10a: Prepared following the general procedure for the Passerini reaction (ramp time: 76 s) using isobutyraldehyde (**9a**, 0.033 g, 0.46 mmol), acid **8** (0.125 g, 0.330 mmol), *tert*-butyl isocyanoacetate (**3b**,

0.065 g, 0.46 mmol) in THF (0,63 mL) to yield product **10a** (0.136 g, 0,230 mmol, 70%) after column chromatography ($CH_2Cl_2 \rightarrow$ 2% MeOH/CH_2Cl_2) as a white foam. R_f (4% MeOH/CH_2Cl_2) = 0.32. IR 3429, 2978, 1751, 1654, 1161 cm^{-1}. ^1H NMR (300 MHz, $CDCl_3$, presence of rotamers): δ 7.38-7.17 (m, 5H), 7.07 (br s, 1H), 5.61 and 5.52 (2 br s, 1H), 5.15 (br d, *J* = 3.6 Hz, 1H), 4.65 (br s, 2H), 4.08-3.94 (m, 7H), 3.82 (dd, *J* = 4.9 and 17.8 Hz, 1H), 2.39-2.27 (m, 1H), 1.44 (s, 9H), 1.42 (s, 9H), 0.97 (d, *J* = 6.8 Hz, 6H). ^{13}C NMR-APT (75.46 MHz, $CDCl_3$, presence of rotamers): δ 170.5 (C=O), 169.4 (C=O), 169.2 (C=O), 168.8 (C=O), 168.6 (C=O), 156.1 (C=O), 134.8 (C), 129.1 (CH), 128.7 (CH), 128.4 (CH), 128.1 (CH), 126.6 (CH), 82.1 (C), 80.0 (C), 78.7 (CH), 51.7 (CH_2), 49.8 (CH_2), 42.2 (CH_2), 42.1 (CH_2), 41.5 (CH_2), 30.6 (CH), 28.3 (CH_3), 28.0 (CH_3), 18.7 (CH_3), 16.7 (CH_3). HRMS (ESI): *m/z*: calc. for $[M+Na]^+$ $C_{29}H_{44}N_4O_9Na$ 615.3006; found: 615.3010.

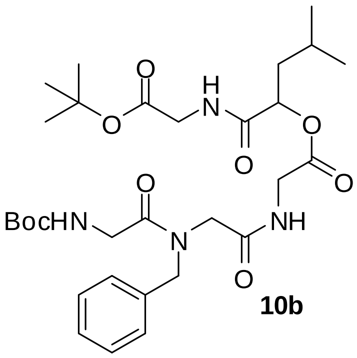

10b

Acyclic depsipeptoid 10b: Prepared following the general procedure for the Passerini reaction (ramp time: 61 s) using isovaleraldehyde (**9b**, 0.041 g, 0.48 mmol), acid **8** (0.130 g, 0.343 mmol), *tert*-butyl isocyanoacetate (**3b**, 0.068 g, 0.48 mmol) in THF (0,34 mL) to yield product **10b** (0.137 g, 0,226 mmol, 66%) after column chromatography ($CH_2Cl_2 \rightarrow$ 2% MeOH/CH_2Cl_2) as a white foam. R_f (4% MeOH/CH_2Cl_2) = 0.32. IR 3341, 2978, 2935, 1748, 1664, 1536, 1384, 1162 cm^{-1}. ^1H NMR (300 MHz, $CDCl_3$, presence of rotamers): δ 7.37-7.10 (m, 6H), 6.99 (br t, *J* = 5.1 Hz, 1H), 5.20 (t, *J* = 8.2 Hz, 1H), 4.60 (d, *J* = 5.0 Hz, 1H), 4.57 (s, 1H), 4.00-3.96 (m, 5H), 3.94-3.84 (m, 2H), 3.81-3.71 (m, 1H), 1.74-1.55 (m, 3H), 1.37 (s, 9H), 1.35 (s, 9H), 0.86 (d, *J* = 5.9 Hz, 3H), 0.84 (d, *J* = 5.9 Hz, 3H). ^{13}C NMR (75.46 MHz, $CDCl_3$, presence of rotamers): δ 170.4 (C=O), 170.2 (C=O), 169.3 (C=O), 168.8 (C=O), 168,7 (C=O), 156.0 (C=O), 134.9 (C), 129.1 (CH), 128.7 (CH), 128.4 (CH), 128.1 (CH), 126.7 (CH), 82.2 (C), 80.0 (C), 73.4 (CH), 51.6 (CH_2), 49.7 (CH_2), 42.1 (CH_2), 41.6 (CH_2), 41.4 (CH_2), 40.6 (CH_2), 28.3 (CH_3), 28.0 (CH_3), 24.4 (CH), 23.0 (CH_3), 21.6 (CH_3). HRMS (ESI): *m/z*: calc. for $[M+Na]^+$ $C_{30}H_{46}N_4O_9Na$ 629.3162; found: 629.3186.

General procedure for the deprotection:

A solution of the acyclic depsipeptoid **10a** or **10b** in CH_2Cl_2 was treated with trifluoroacetic acid (TFA) at 0 °C under nitrogen atmosphere. After stirring for 40 h at room temperature, the solvent was removed under vacuum, diluted and concentrated in vacuum three times. The residue was used without further purification in the following reaction.

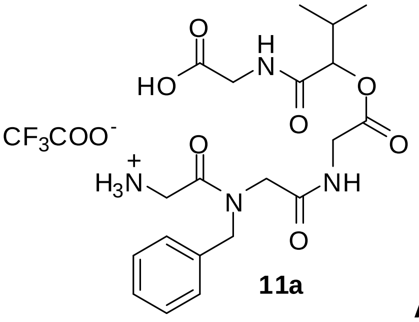

11a

Amino acid as TFA salt 11a: Prepared following the general procedure for the deprotection using acyclic depsipeptoid **10a** (0.114 g, 0.192 mmol) with TFA (0.97 mL) and CH_2Cl_2 (3.9 mL), yielding amino acid as TFA salt **11a** in quantitative yield as a viscous yellow oil, which was used in the next step without further purification. ^{1}H NMR (300 MHz, CD_3OD, presence of rotamers): δ 7.43-7.26 (m, 5H), 5.00 (d, J = 4.6 Hz, 1H), 4.67 (d, J = 15.1 Hz, 1H), 4.60 (d, J = 15.1 Hz, 1H), 4.15-3.92 (m, 8H), 2.32-2.20 (m, 1H), 1.01 (d, J = 6.8 Hz, 6H). ^{13}C NMR (75.46 MHz, CD_3OD, presence of rotamers): δ 172.5, 172.2, 171.2, 170.6, 168.6, 163.1, 137.2, 130.2, 129.7, 129.4, 128.9, 128.2, 79.8, 52.3, 51.2, 42.1, 41.5, 41.2, 32.0, 19.1, 17.3.

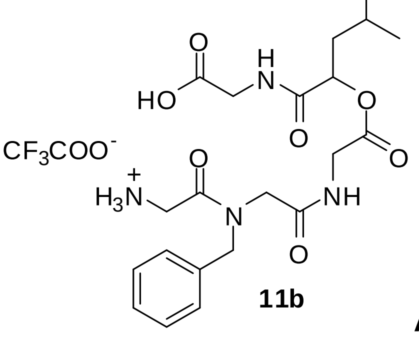

11b

Amino acid as TFA salt 11b: Prepared following the general procedure for the deprotection using acyclic depsipeptoid **10b** (0.059 g, 0.097 mmol) with TFA (0.49 mL) and CH_2Cl_2 (2.0 mL), yielding amino acid as TFA salt **11b** in quantitative yield as a viscous yellow oil, which was used in the next step without

further purification. ^{1}H NMR (300 MHz, CD_3OD, presence of rotamers): δ 7.42-7.28 (m, 5H), 5.22-5.17 (m, 1H), 4.66 (d, J = 14,6 Hz, 1H), 4,60 (d, J = 14.6 Hz, 1H), 4.14-3.91 (m, 8H), 1.94-1.61 (m, 2H), 1.56-1.51 (m, 1H), 0.95 (d, J = 6.4 Hz, 6H). ^{13}C NMR (75.46 MHz, CD_3OD, presence of rotamers): δ 178.5, 173.3, 173.0, 172.7, 170.5, 168.7, 137.2, 130.2, 129.8, 129.4, 128.9, 128.2, 71.3, 52.4, 51.2, 44.6, 42.1, 41.9, 41.5, 25.6, 23.5, 22.0.

General procedure for macrocyclization via intramolecular Ugi reaction

A solution of the amino acid TFA salt **11a** or **11b** (0.20 mmol) in 50 mL of MeOH at room temperature was added via a syringe pump to a solution of paraformaldehyde (0.40 mmol), isocyanide (0.80 mmol), triethylamine (0.20 mmol), anhydrous sodium sulfate (2.25 g) in methanol (200 mL) at a rate of 0.6 mL/h (addition time: 83 h; concentration: 0.80 mmol/L). After the addition was complete (~ 4 days), the reaction mixture was stirred for further 24 h, filtered, concentrated in vacuum, diluted in CH_2Cl_2, washed with saturated NaCl solution, dried with Na_2SO_4, the solvent was removed and the residue purified by column chromatography.

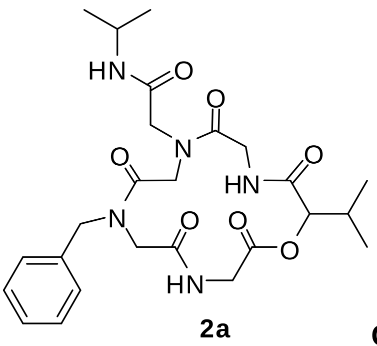

2a

Cyclic pentadepsipeptoid 2a: Prepared following the general procedure for macrocyclization using the amino acid TFA salt **11a** (0.106 g, 0.193 mmol), paraformaldehyde (**4**, 0.012 g, 0.38 mmol), isopropyl isocyanide (**12a**, 0.053 g, 0.77 mmol), triethylamine (0.020 g, 0.19 mmol) and anhydrous sodium sulfate (2.17 g) to furnish cyclic pentadepsipeptoid **2a** (0.033 g, 33%) after column chromatography (CH_2Cl_2 → 4% MeOH/CH_2Cl_2) as a white solid (m.p. = 124-126 °C). R_f (8% MeOH/CH_2Cl_2) = 0.26. IR 3291, 3083, 2970, 2934, 1748, 1667, 1550, 1467, 1226, 1000, 956, 737, 702 cm^{-1}. ^{1}H NMR (300 MHz, DMSO-d_6, presence of rotamers): δ 9.34 and 8.79 (2br s, 1H), 7.86-7.71 (m, 1H), 7.42-7.27 (m, 5H), 5.21-3.42 (m, 14H), 2.40-2.30 and 2.15-2.00 (2m, 1H), 1.06, 0.99 and 0.97 (3d, J = 6.6 Hz, 6H), 0.93, 0.87 and

0.79 (3d, *J* = 6.6 Hz, 6H). ^{13}C NMR (75.46 MHz, DMSO-d$_6$, presence of rotamers): δ 171.6, 170.0, 169.3, 168.4, 167.4, 166.6, 136.3, 128.7, 128.3, 127.8, 127.6, 127.2, 77.3, 54.7, 53.1, 51.8, 50.7, 48.6, 44.7, 41.9, 30.5, 22.2, 18.4, 16.8, 15.7. HRMS (ESI): *m/z*: calc. for $[M+Na]^+$ $C_{25}H_{35}N_5O_7Na$ 540.2434; found: 540.2431.

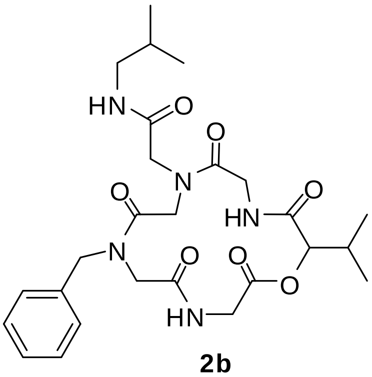

2b

Cyclic pentadepsipeptoid 2b: Prepared following the general procedure for macrocyclization using the amino acid TFA salt **11a** (0.105 g, 0.191 mmol), paraformaldehyde (**4**, 0.011 g, 0.38 mmol), isobutyl isocyanide (**12b**, 0.063 g, 0.76 mmol), triethylamine (0.019 g, 0.19 mmol) and anhydrous sodium sulfate (2.12 g) to furnish cyclic pentadepsipeptoid **2b** (0.040 g, 40%) after column chromatography (CH_2Cl_2 → 4% MeOH/CH_2Cl_2) as a white solid. R_f (8% MeOH/CH_2Cl_2) = 0.26. IR 3295, 3088, 2963, 2934, 2874, 1748, 1668, 1548, 1469, 1281, 1226, 1004, 956, 737, 701 cm^{-1}. ^{1}H NMR (300 MHz, DMSO-d$_6$, presence of rotamers): δ 9.34 and 8.79 (2br s, 1H), 8.06 and 7.94 (2 br t, *J* = 5.9 Hz, 1H), 7.41-7.27 (m, 5H), 5.19-3.38 (m, 13H), 2.88-2.73 (m, 2H), 2.44-2.25 and 2.14-2.03 (m, 1H), 1.67-1.56 (m, 1H), 0.86 (d, *J* = 7.0 Hz, 6H), 0.80 and 0.77 (2d, *J* = 6.6 Hz, 6H). ^{13}C NMR (75.46 MHz, DMSO-d$_6$, presence of rotamers): δ 171.5, 170.1, 169.7, 169.1, 168.3, 167.6, 136.2, 128.6, 128.3, 127.7, 127.5, 127.1, 77.3, 51.8, 51.5, 50.5, 50.1, 48.5, 45.8, 41.9, 30.4, 27.9, 19.9, 18.3, 16.7. HRMS (ESI): *m/z*: calc. for $[M+Na]^+$ $C_{26}H_{37}N_5O_7Na$ 554.2591; found: 554.2588.

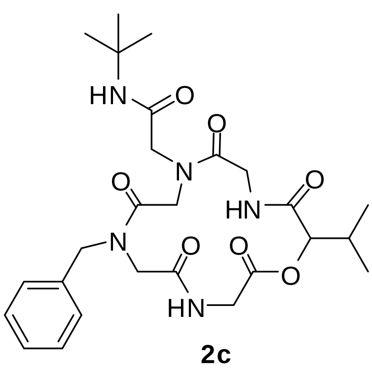

2c **Cyclic pentadepsipeptoid 2c**: Prepared following the general procedure for macrocyclization using the amino acid TFA salt **11a** (0.111 g, 0.202 mmol), paraformaldehyde (**4**, 0.012 g, 0.40 mmol), *tert*-butyl isocyanide (**12c**, 0.067 g, 0.81 mmol), triethylamine (0.020 g, 0.20 mmol) and anhydrous sodium sulfate (2.25 g) to furnish cyclic pentadepsipeptoid **2c** (0.053 g, 49%) after column chromatography (CH_2Cl_2 → 4% MeOH/CH_2Cl_2) as a white solid (m.p. = 202-204 ºC). R_f (8% MeOH/CH_2Cl_2) = 0.31. IR 3340, 3295, 3078, 2969, 1765, 1672, 1640, 1564, 1459, 1398, 1342, 1274, 1221, 1000, 960, 710 cm^{-1}. ^1H NMR (300 MHz, DMSO-d_6, presence of rotamers): δ 9.34 and 8.76 (2 br s, 1H), 7.62 and 7.54 (2br s, 1H), 7.42-7.22 (m, 6H), 5.20-3.38 (m, 13H), 2.44-2.32 and 2.22-2.02 (2m, 1H), 1.20 and 1.18 (2s, 9H), 0.87 and 0.79 (2d, J = 6.3 Hz, 6H). ^{13}C NMR (75.46 MHz, DMSO-d_6, presence of rotamers): δ 171.6, 170.0, 169.5, 169.2, 168.3, 166.9, 136.4, 128.7, 128.3, 127.7, 127.0, 77.3, 53.8, 51.8, 51.1, 50.7, 50.0, 48.7, 42.3, 30.5, 28.3, 18.4, 16.8. HRMS (ESI): *m/z*: calc. for $[M+Na]^+$ $C_{26}H_{37}N_5O_7Na$ 554.2591; found: 554.2579.

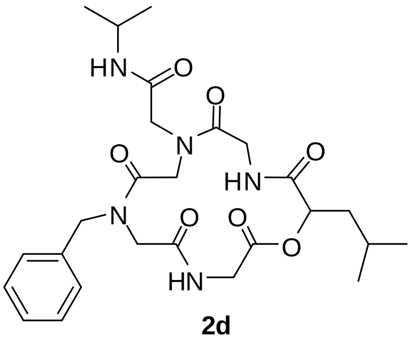

2d **Cyclic pentadepsipeptoid 2d**: Prepared following the general procedure for macrocyclization using the amino acid TFA salt **11b** (0.109 g, 0.193 mmol), paraformaldehyde (**4**, 0.012 g, 0.39 mmol), isopropyl isocyanide (**12a**, 0.053 g, 0.77 mmol), triethylamine (0.020 g, 0.193 mmol) and anhydrous sodium sulfate (2.16 g) to furnish cyclic pentadepsipeptoid **2d** (0.036 g, 35%) after column chromatography (CH_2Cl_2 → 4% MeOH/CH_2Cl_2) as a white solid (m.p. = 224-226 ºC). R_f (8% MeOH/CH_2Cl_2) = 0.28. IR 3317, 3086, 2963, 2937, 2872, 1752, 1673, 1655, 1544,

1471, 1227, 971, 734 cm^{-1}. ^1H NMR (300 MHz, DMSO-d_6, presence of rotamers): δ 9.29 and 8.68 (2br s, 1H), 7.83-7.72 (m, 1H), 7.41-7.27 (m, 6H), 5.32-3.38 (m, 14 H), 1.74-1.57 (m, 3H), 1.03-0.86 (m, 12H). ^{13}C NMR (75.46 MHz, DMSO-d_6, presence of rotamers): δ 171.3, 169.9, 169.5, 169.0, 168.6, 166.6, 136.3, 128.7, 128.3, 127.7, 127.0, 71.9, 51.8, 51.2, 50.5, 50.0, 48.7, 44.8, 41.8, 40.9, 23.6, 22.9, 22.2 (2C), 21.4. HRMS (ESI): *m/z*: calc. for $[M+Na]^+$ $C_{26}H_{37}N_5O_7Na$ 554.2591; found: 554.2598.

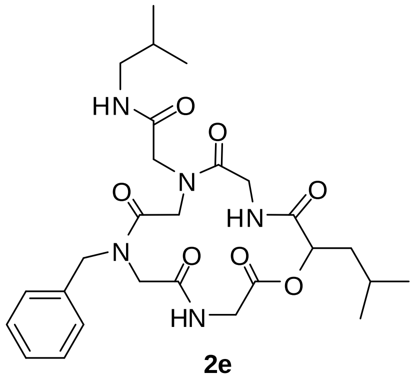

2e

Cyclic pentadepsipeptoid 2e: Prepared following the general procedure for macrocyclization using the amino acid TFA salt **11b** (0.098 g, 0.173 mmol), paraformaldehyde(**4**, 0.010 g, 0.35 mmol), isobutyl isocyanide (**12b**, 0.058 g, 0.693 mmol), triethylamine (0.018 g, 0.173 mmol) and anhydrous sodium sulfate (1.93 g) to furnish cyclic pentadepsipeptoid **2e** (0.035 g, 37%) after column chromatography (CH_2Cl_2 → 4% MeOH/CH_2Cl_2) as a white solid (m.p. = 194-196 ºC). R_f (8% MeOH/CH_2Cl_2) = 0.28. IR 3311, 3266, 3092, 2958, 2933, 2871, 1752, 1676, 1648, 1538, 1471, 1444, 1232, 737, 699 cm^{-1}. ^1H NMR (300 MHz, DMSO-d_6, presence of rotamers): δ 9.28 and 8.70 (2br s, 1H), 7.96 and 7.73 (2br t, 1H), 7.41-7.25 (m, 5H), 7.07 (br s, 1H), 5.31-3.40 (m, 13H), 2.96-2.73 (m, 2H), 1.74-1.56 (m, 4H), 0.88-0.83 (m, 6H), 0.78 and 0.76 (2d, J = 6.7 Hz, 6H). ^{13}C NMR (75.46 MHz, DMSO-d_6, presence of rotamers): δ 171.2, 169.9, 169.6, 169.0, 168.6, 167.6, 137.0, 128.6, 128.3, 127.6, 127.1, 71.9, 53.0, 51.8, 51.1, 50.4, 48.6, 45.8, 41.9, 40.8, 28.0, 23.6, 22.8, 21.3, 20.0. HRMS (ESI): *m/z*: calc. for $[M+Na]^+$ $C_{27}H_{39}N_5O_7Na$ 568.2747; found: 568.2744.

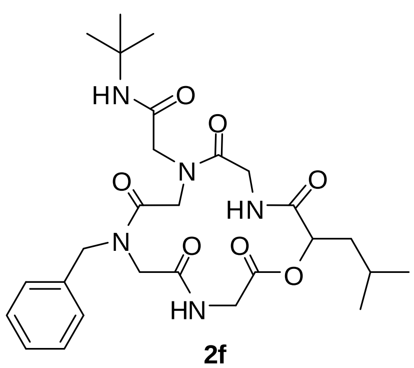

2f

Cyclic pentadepsipeptoid 2f: Prepared following the general procedure for macrocyclization using the amino acid TFA salt **11b** (0.113 g, 0.200 mmol), paraformaldehyde (**4**, 0.012 g, 0.40 mmol), *tert*-butyl isocyanide (**12c**, 0.066 g, 0.80 mmol), triethylamine (0.020 g, 0.20 mmol) and anhydrous sodium sulfate (2.25 g) to furnish cyclic pentadepsipeptoid **2f** (0.036 g, 33%) after column chromatography (CH_2Cl_2 → 4% MeOH/CH_2Cl_2) as a white solid (m.p. = 246-248 ºC). R_f (8% MeOH/CH_2Cl_2) = 0.30. IR 3328, 3264, 3084, 2964, 2933, 1750, 1679, 1650, 1564, 1535, 1472, 1447, 1226, 743, 703 cm^{-1}. ^1H NMR (300 MHz, DMSO-d_6, presence of rotamers): δ 9.30 and 8.65 (2br s, 1H), 7.62 (s, 1H), 7.42-7.27 (m, 5H), 5.32-3.43 (m, 13H), 1.73-1.54 (m, 3H), 1.21 and 1.18 (2s, 9H), 0.87 (d, J = 5.9 Hz, 6H). ^{13}C NMR (75.46 MHz, DMSO-d_6, presence of rotamers): δ 171.4, 169.9, 169.3, 169.0, 168.6, 166.9, 136.3, 128.7, 128.3, 127.6, 127.0, 71.9, 51.8, 51.0, 50.6, 50.0, 48.1, 44.4, 41.8, 40.9, 28.3, 23.6, 22.9, 21.4. HRMS (ESI): *m/z*: calc. for $[M+Na]^+$ $C_{27}H_{39}N_5O_7Na$ 568.2747; found: 568.2744.

References

1. Barreto, A. F. S.; Vercillo, O. E.; Birkett, M. A.; Caulfied, J. C.; Wessjohann, L. A.; Andrade, C. K. Z. *Org. Biom. Chem*. **2011,** *9,* 5024–5027.

2. Barreto, A. F. S.; Vercillo, O. E.; Andrade, C. K. Z. *J. Braz. Chem. Soc*. **2011,** *22,* 462–467.

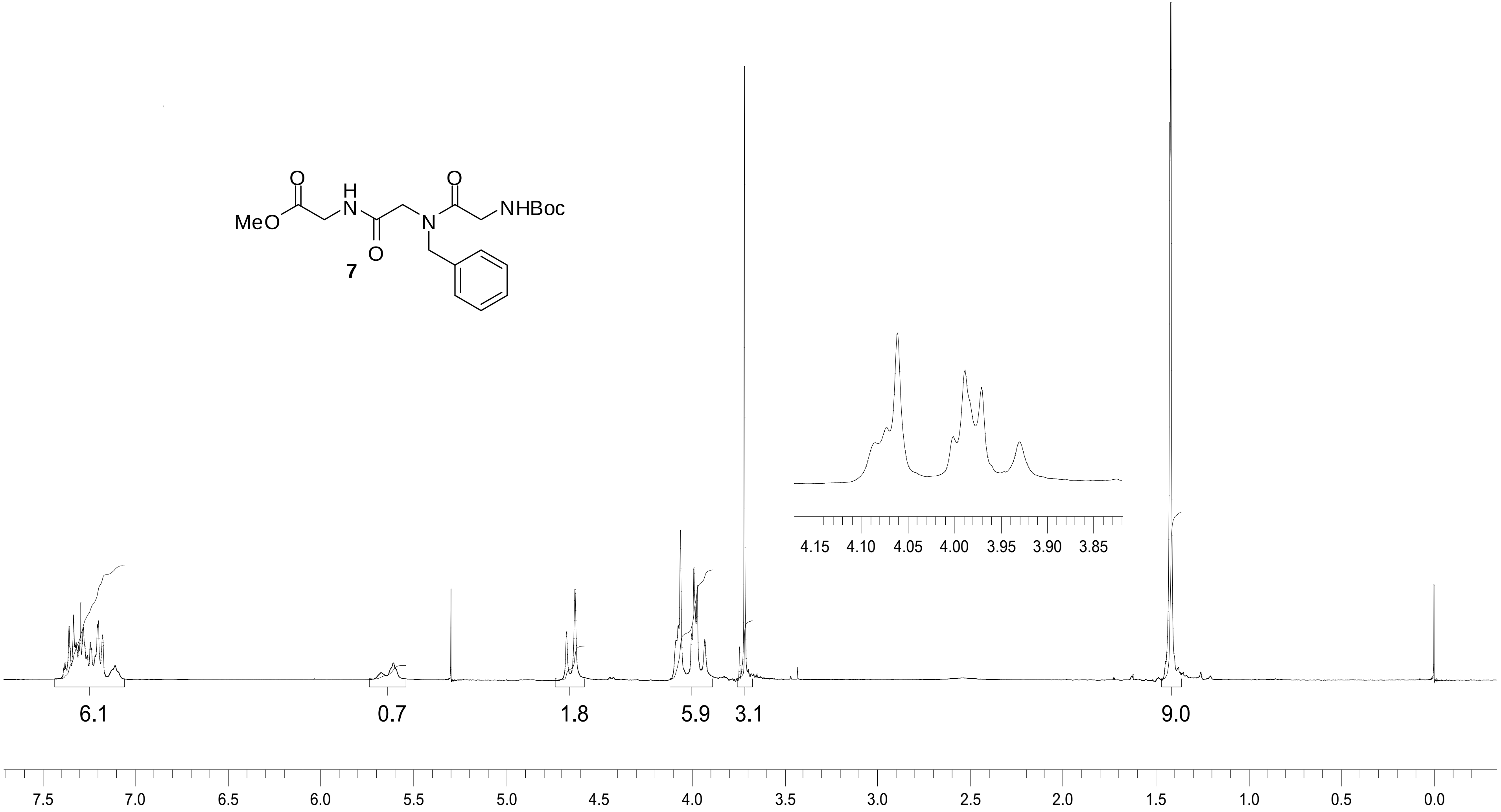

Figure S1: ^{1}H NMR (300 MHz, $CDCl_3$) spectrum of compound **7**.

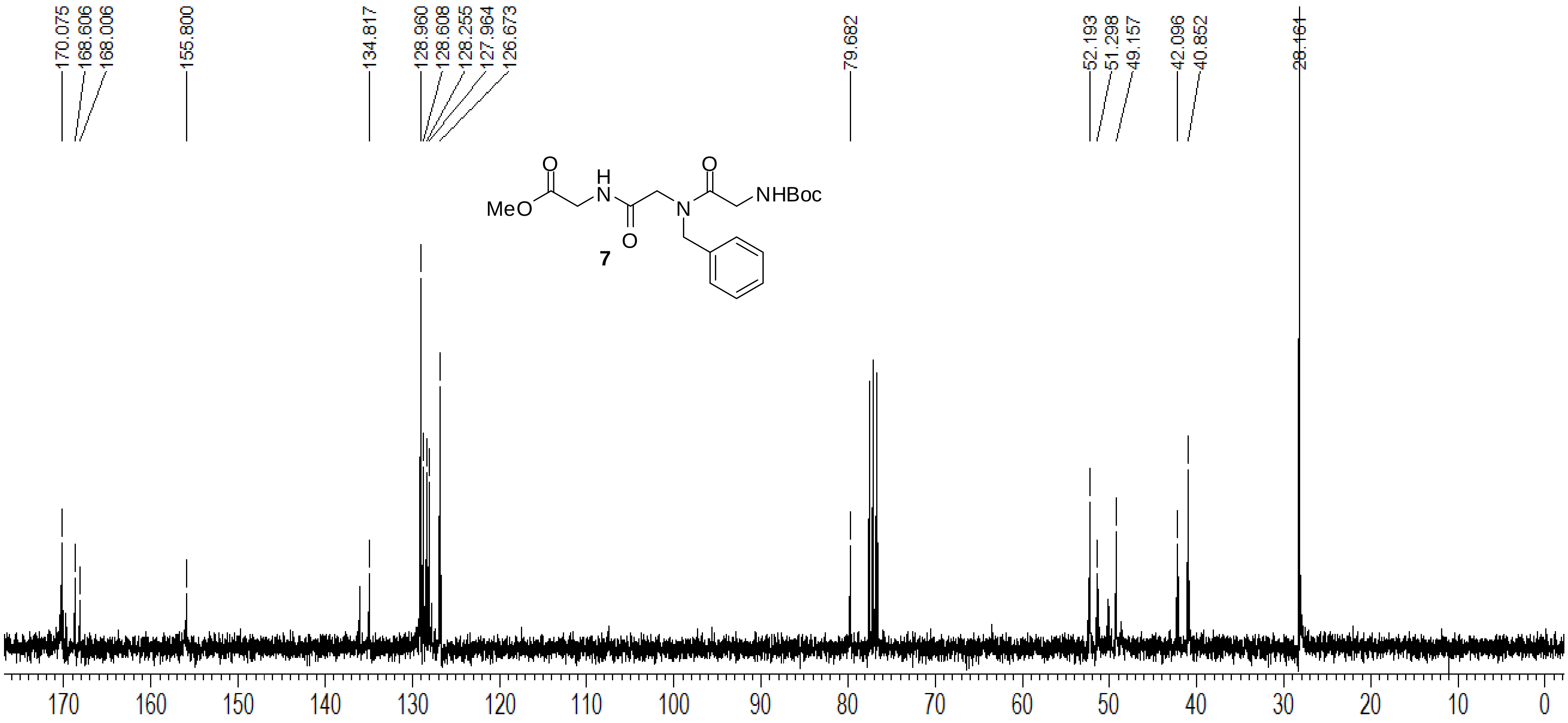

Figure S2: ^{13}C NMR (75.46 MHz, $CDCl_3$) spectrum of compound **7**.

Equipment: MicroTOF **Manufacturer: Bruker Daltonics**

HV = 4500 V; Temp: 180 ºC; Flow: 180 μl/h

ESI⁺ MeOH

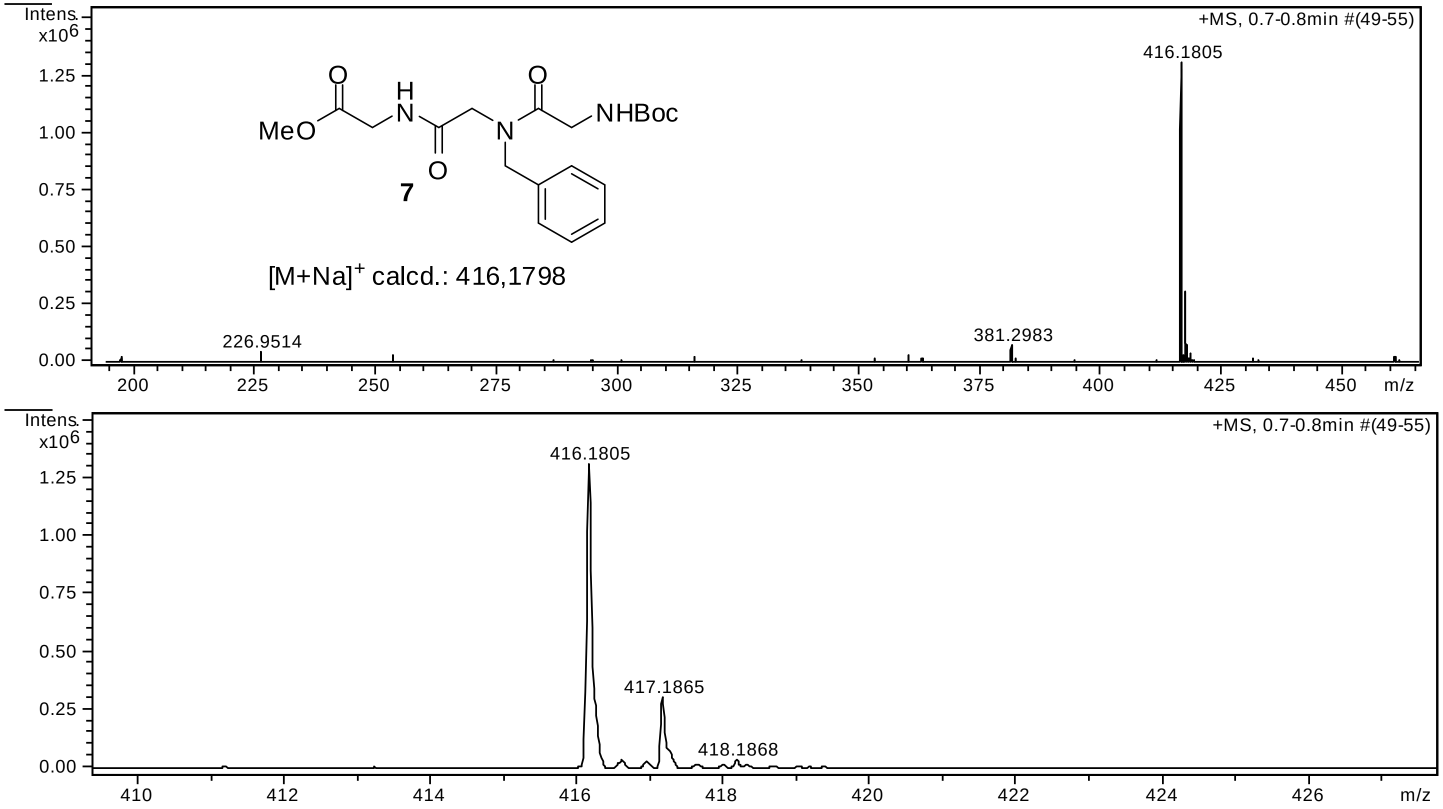

Figure S3: ESI-HRMS of compound **7.**

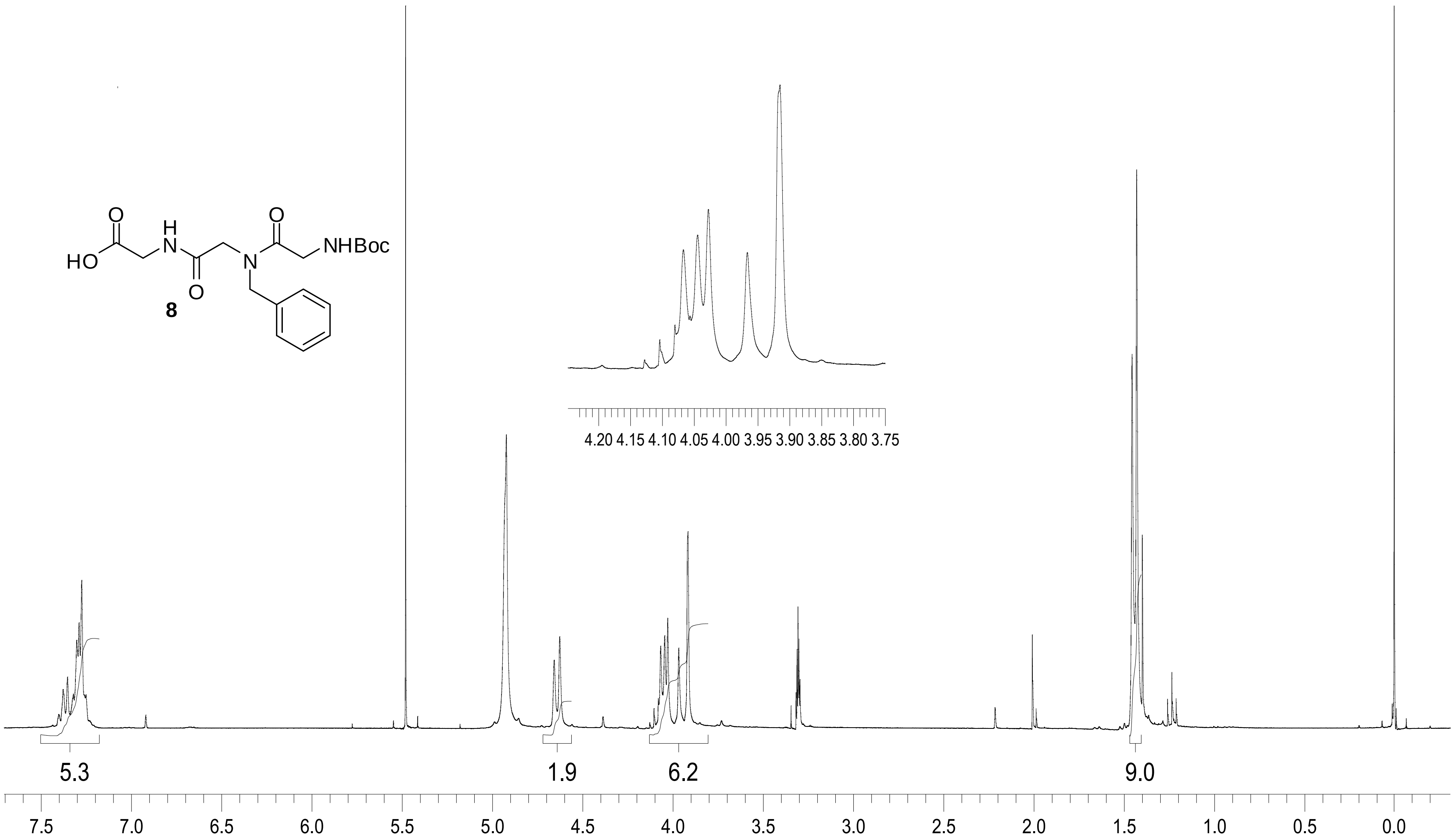

Figure S4: ^{1}H NMR (300 MHz, CD_3OD, presence of rotamers) spectrum of compound **8**.

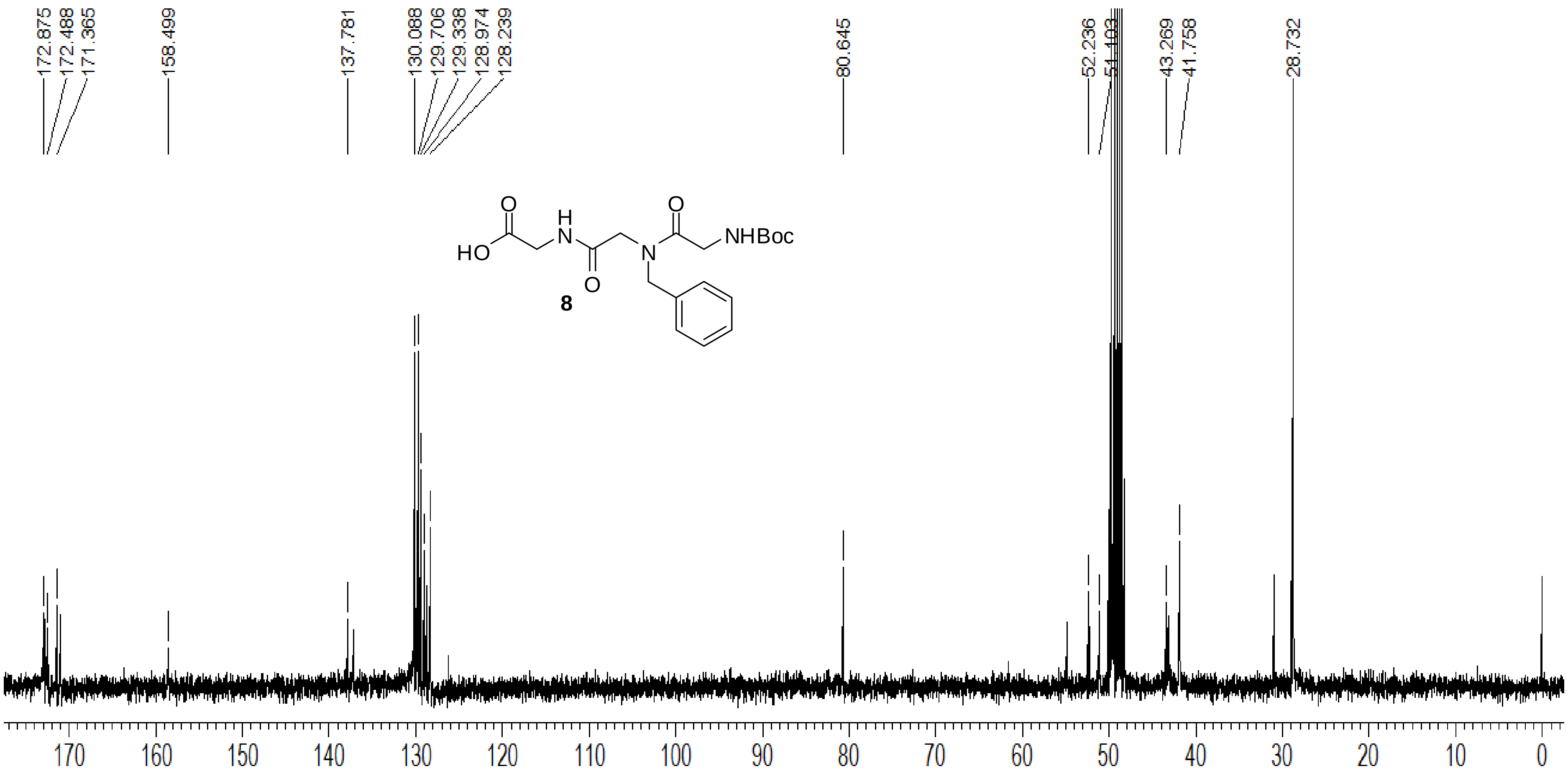

Figure S5: ^{13}C NMR (75.46 MHz, CD_3OD, presence of rotamers) spectrum of compound **8**.

Equipment: MicroTOF **Manufacturer: Bruker Daltonics**

HV = 4500 V; Temp: 180 ºC; Flow: 180 μl/h

ESI$^+$ MeOH

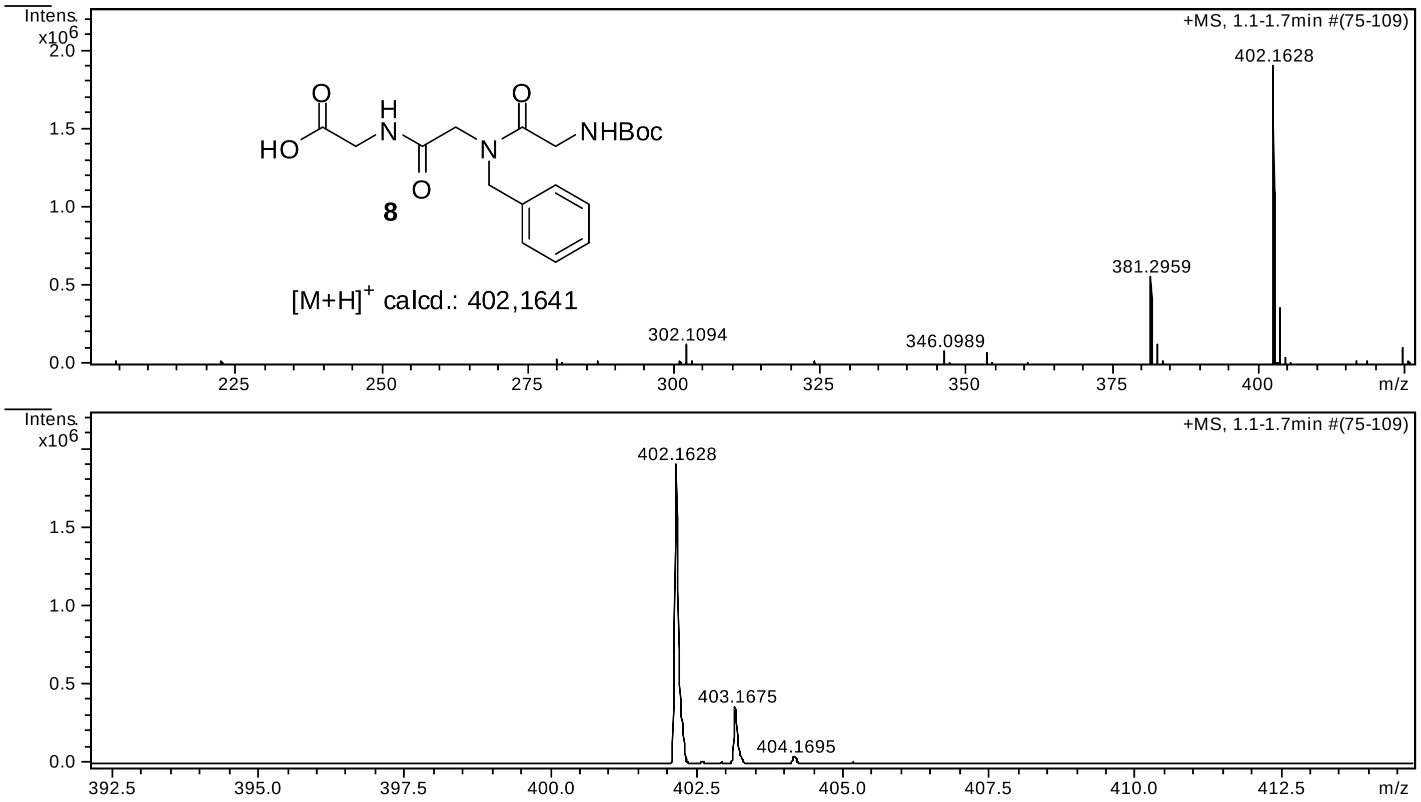

Figure S6: ESI-HRMS of compound **8.**

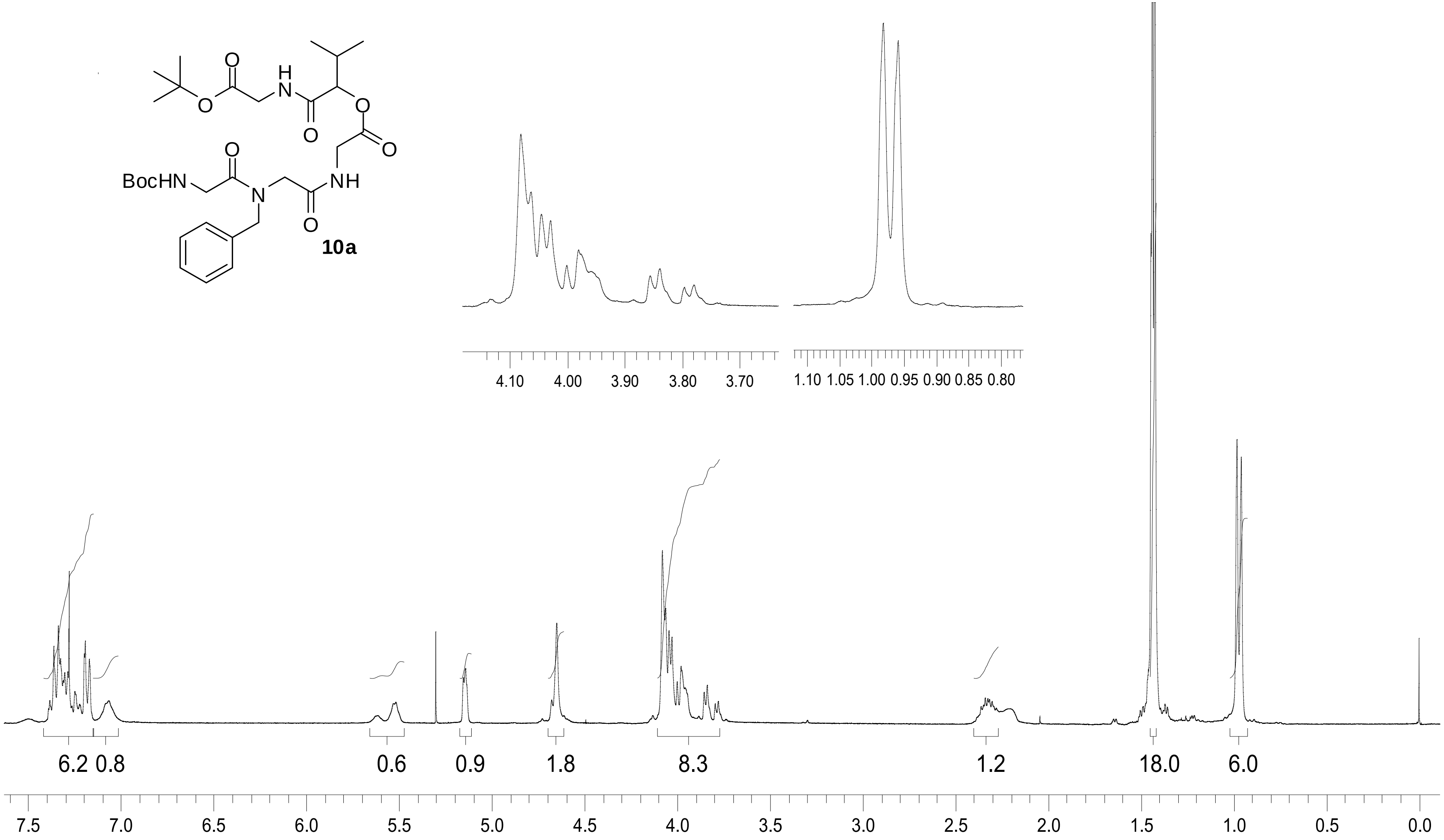

Figure S7: ^{1}H NMR (300 MHz, $CDCl_3$, presence of rotamers) spectrum of compound **10a**.

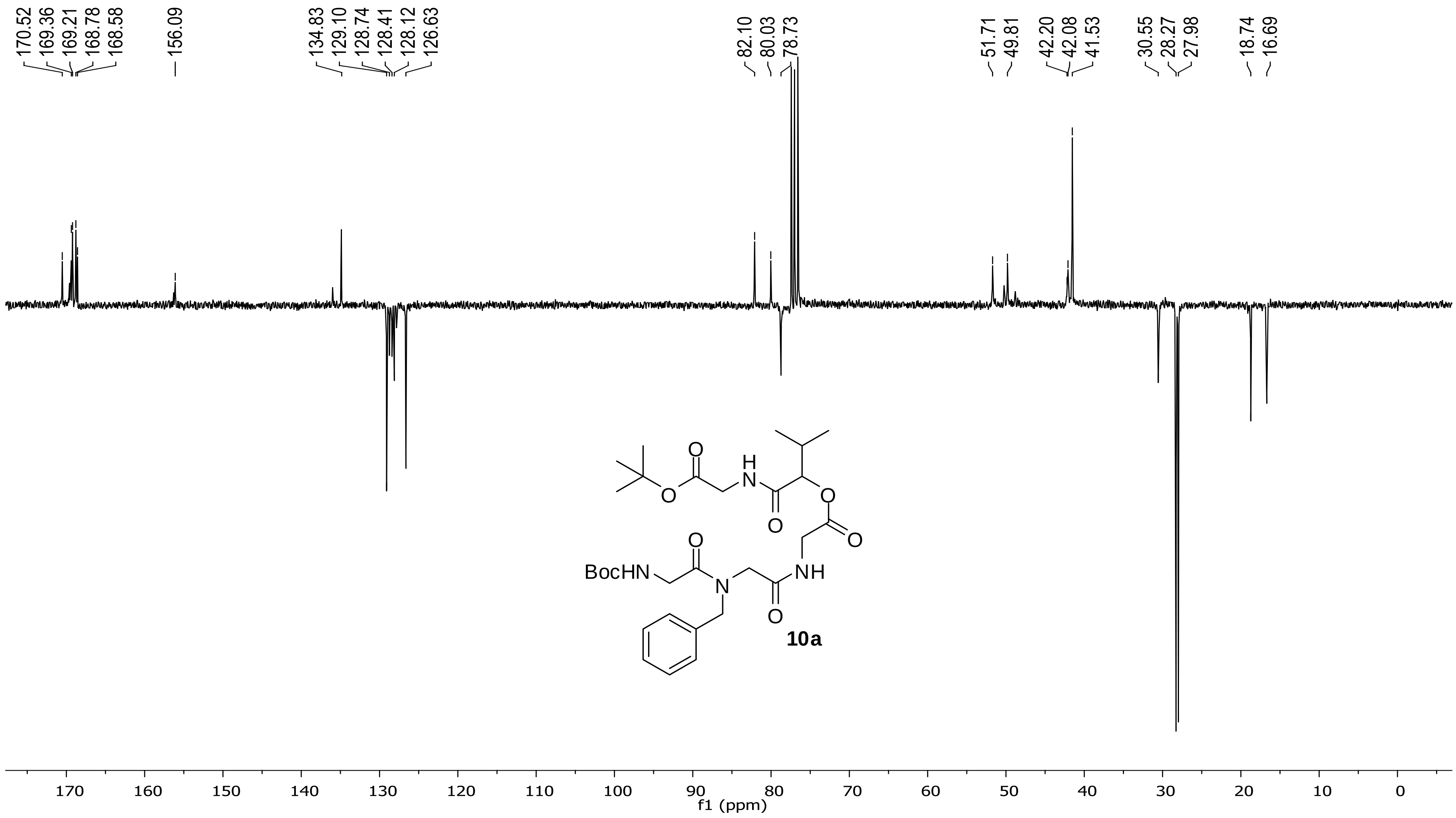

Figure S8: ^{13}C NMR-APT (75.46 MHz, $CDCl_3$, presence of rotamers) spectrum of compound **10a**.

Equipment: MicroTOF **Manufacturer: Bruker Daltonics**

HV = 4500 V; Temp: 180 ºC; Flow: 180 μl/h

ESI$^+$ MeOH

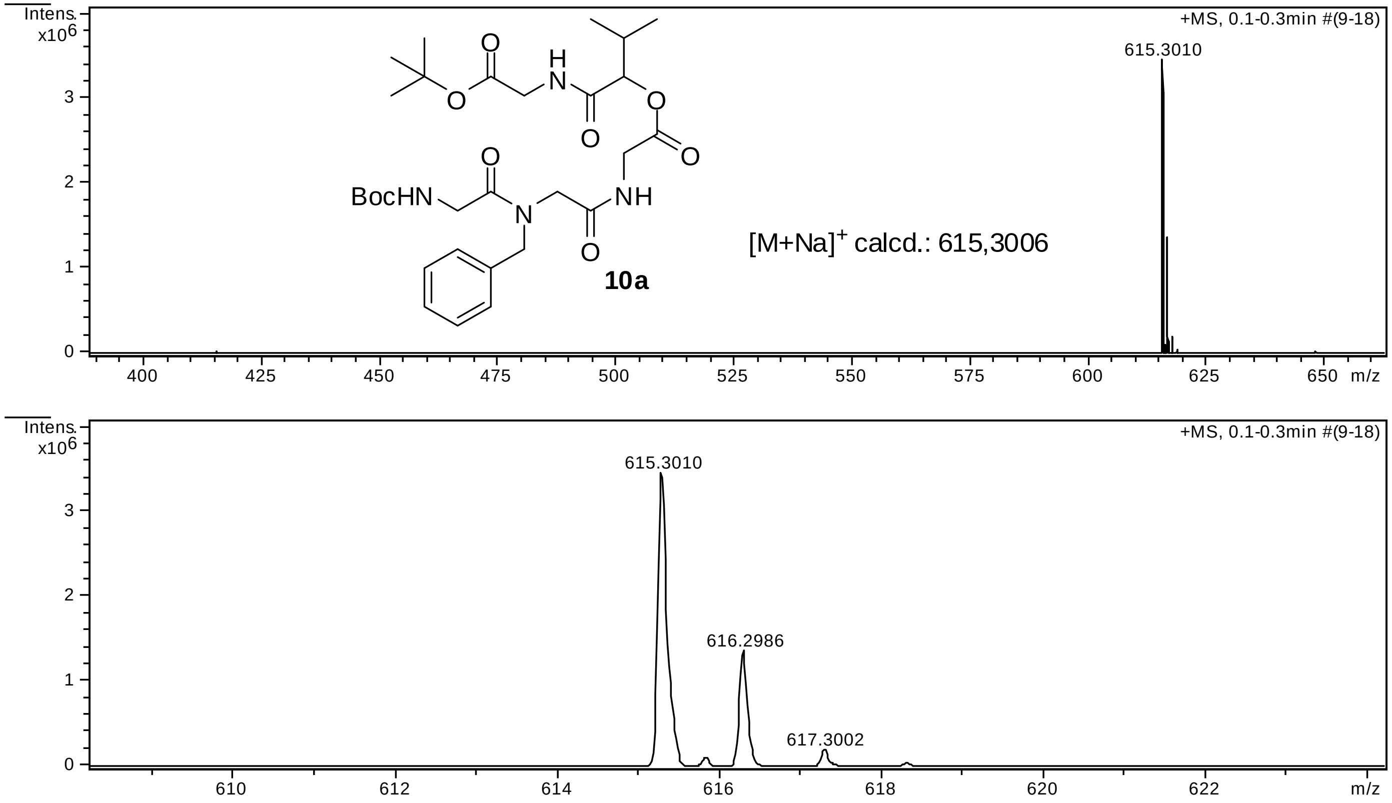

Figure S9: ESI-HRMS of compound **10a.**

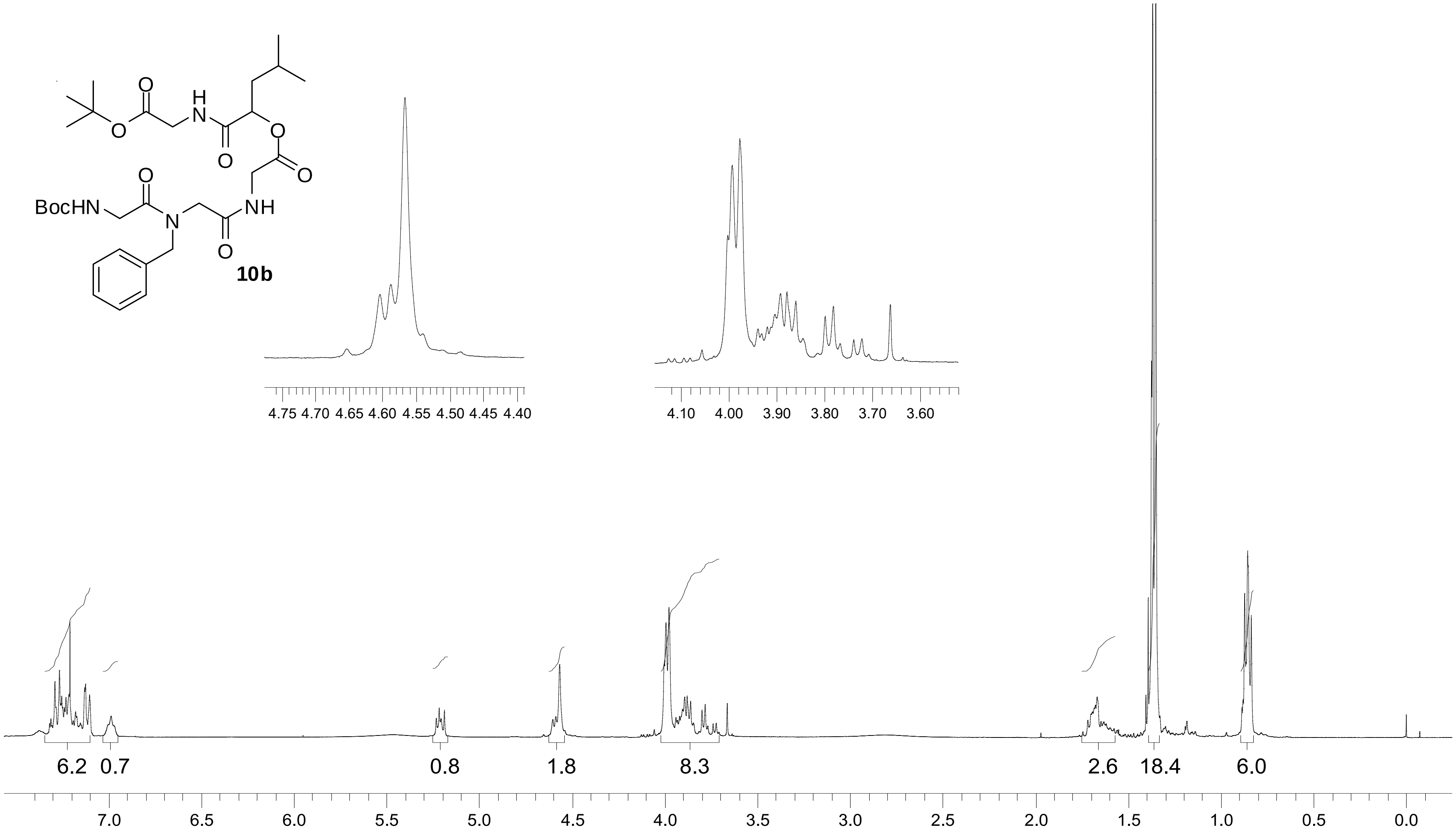

Figure S10: ^{1}H NMR (300 MHz, $CDCl_3$, presence of rotamers) spectrum of compound **10b**.

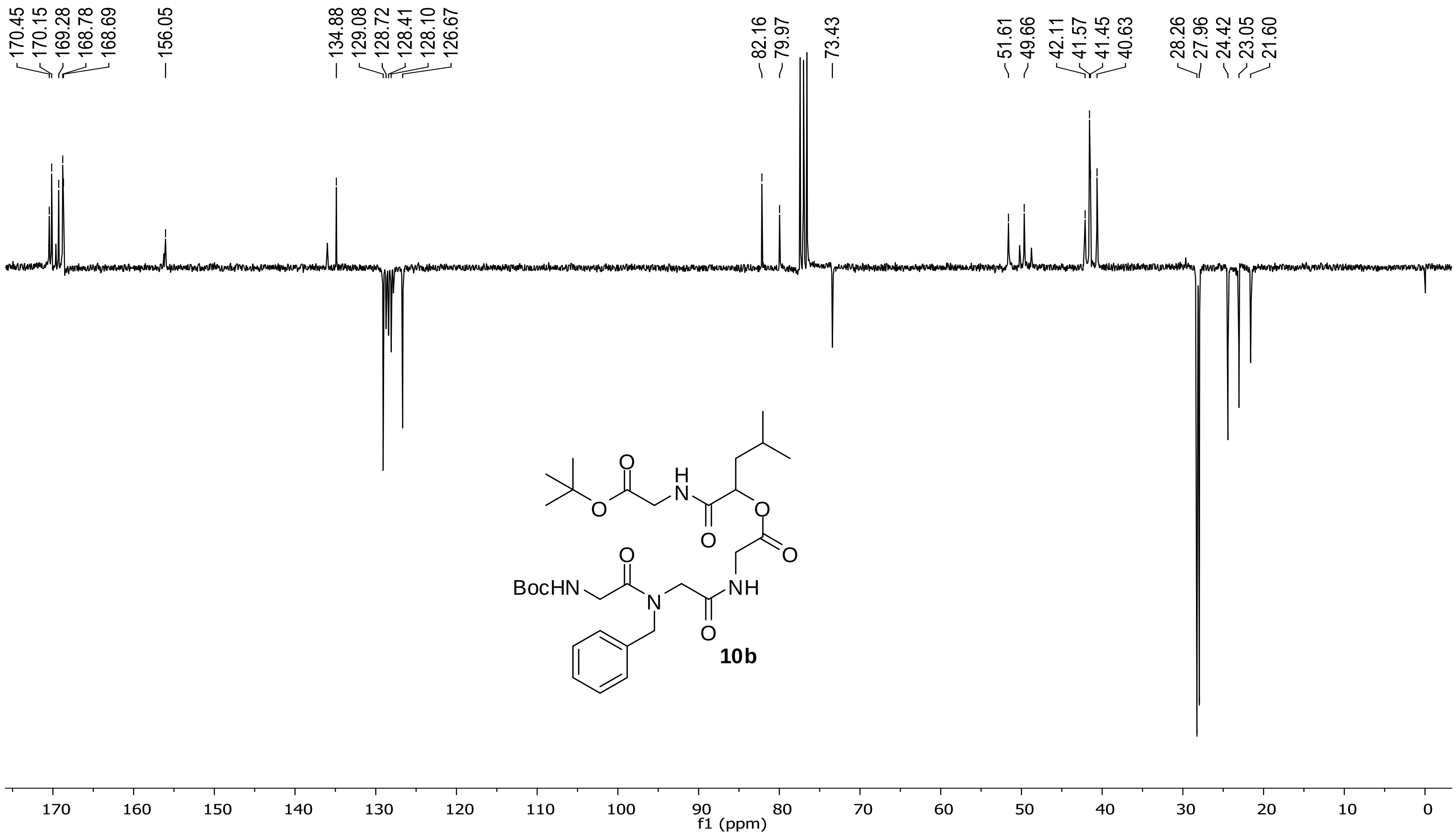

Figure S11: ^{13}C NMR-APT (75.46 MHz, $CDCl_3$, presence of rotamers) spectrum of compound **10b**.

Equipment: MicroTOF **Manufacturer: Bruker Daltonics**

HV = 4500 V; Temp: 180 ºC; Flow: 240 μl/h

ESI^+ ACN 0.1% Formic acid

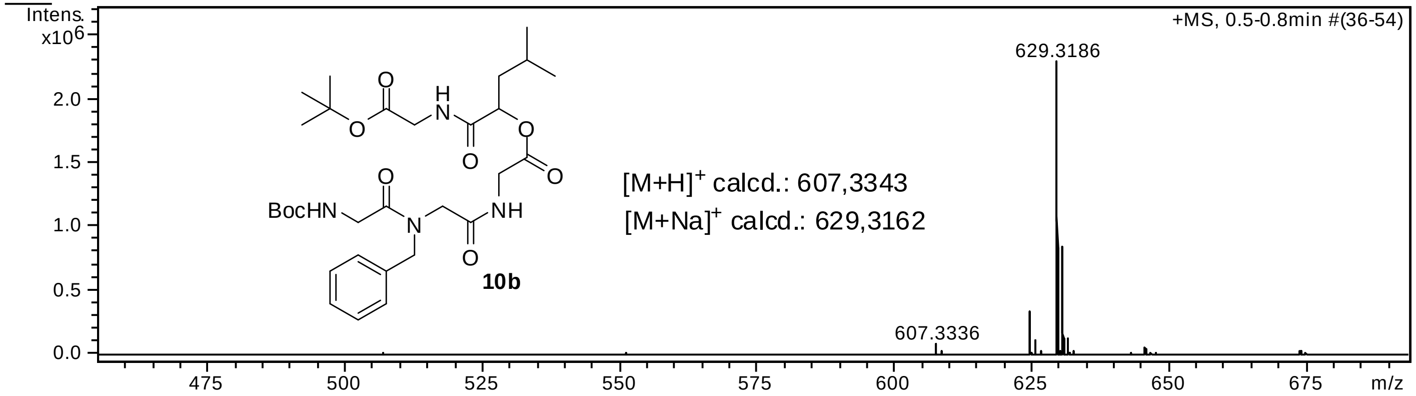

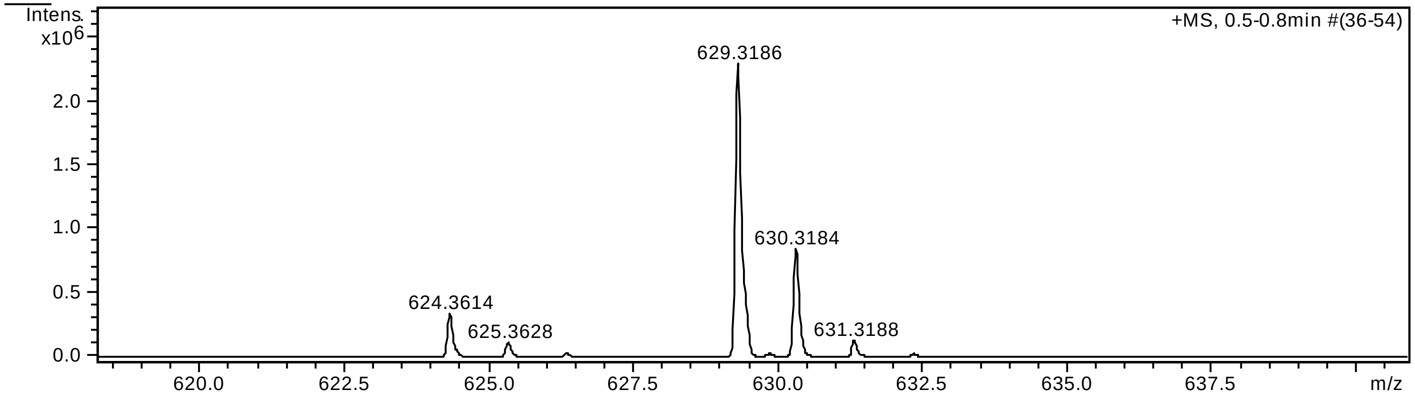

Figure S12: ESI-HRMS of compound **10b.**

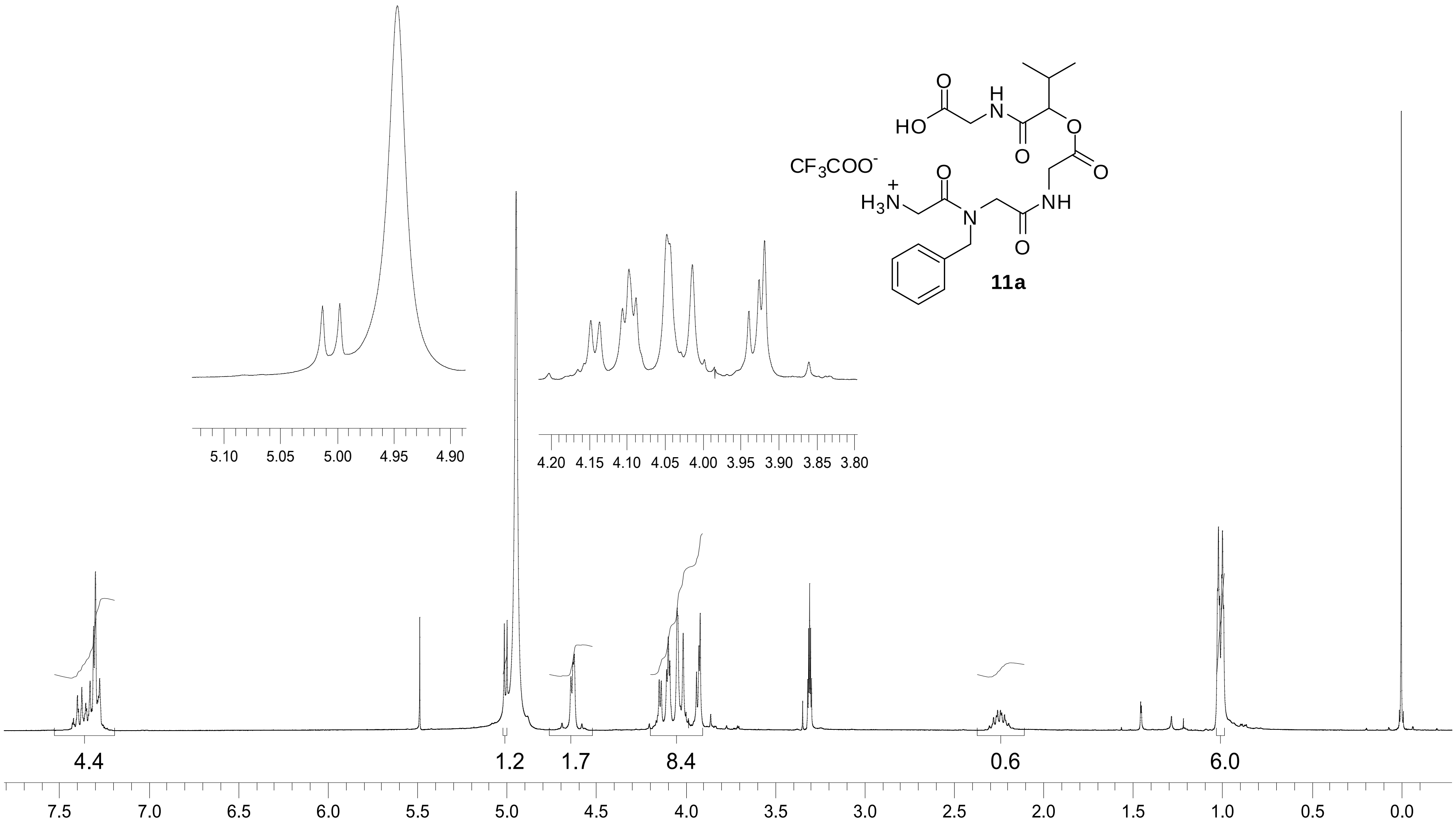

Figure S13: ^{1}H NMR (300 MHz, CD_3OD, presence of rotamers) spectrum of compound **11a**.

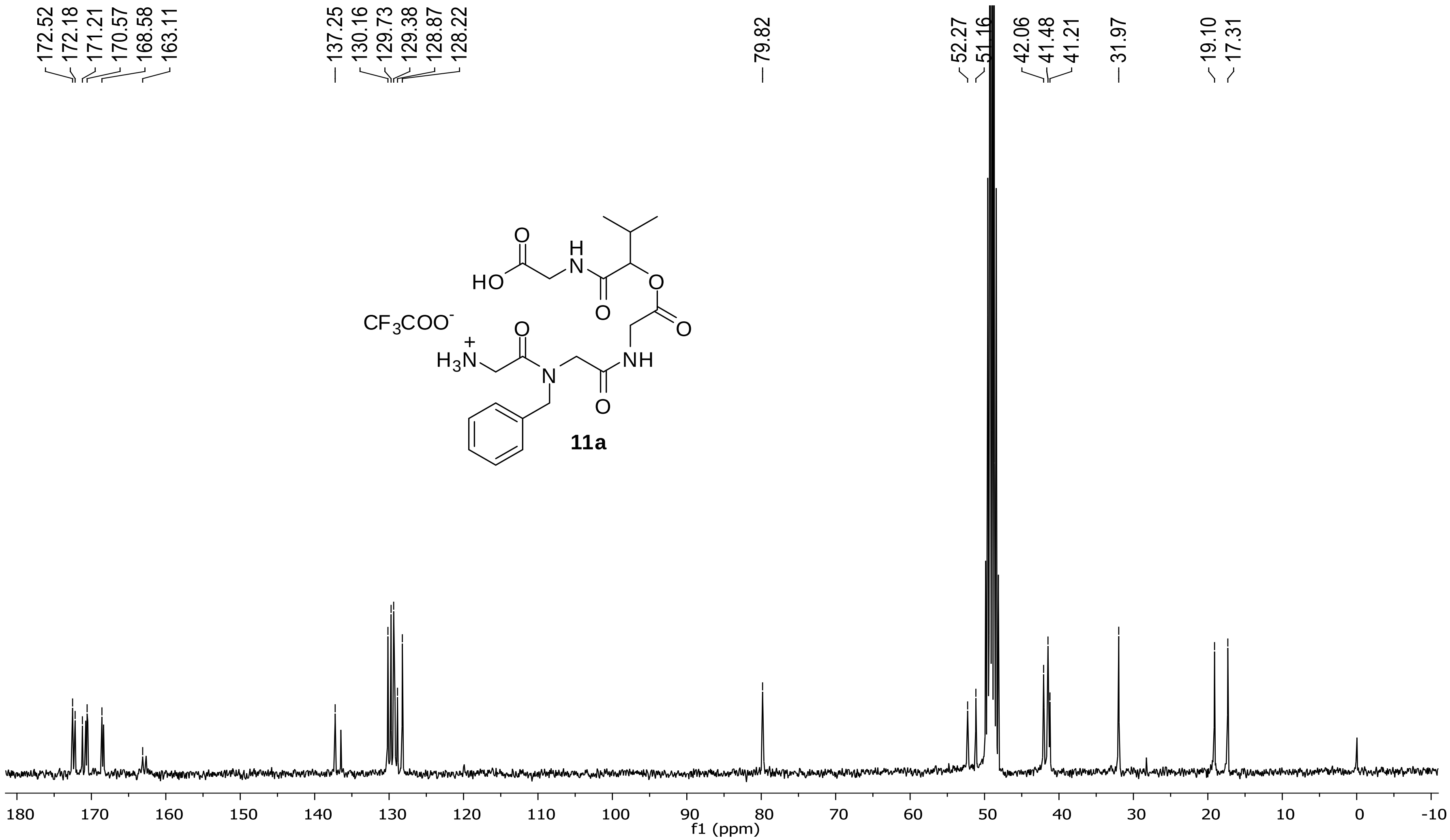

Figure S14: ^{13}C NMR (75.46 MHz, CD_3OD, presence of rotamers) spectrum of compound **11a**.

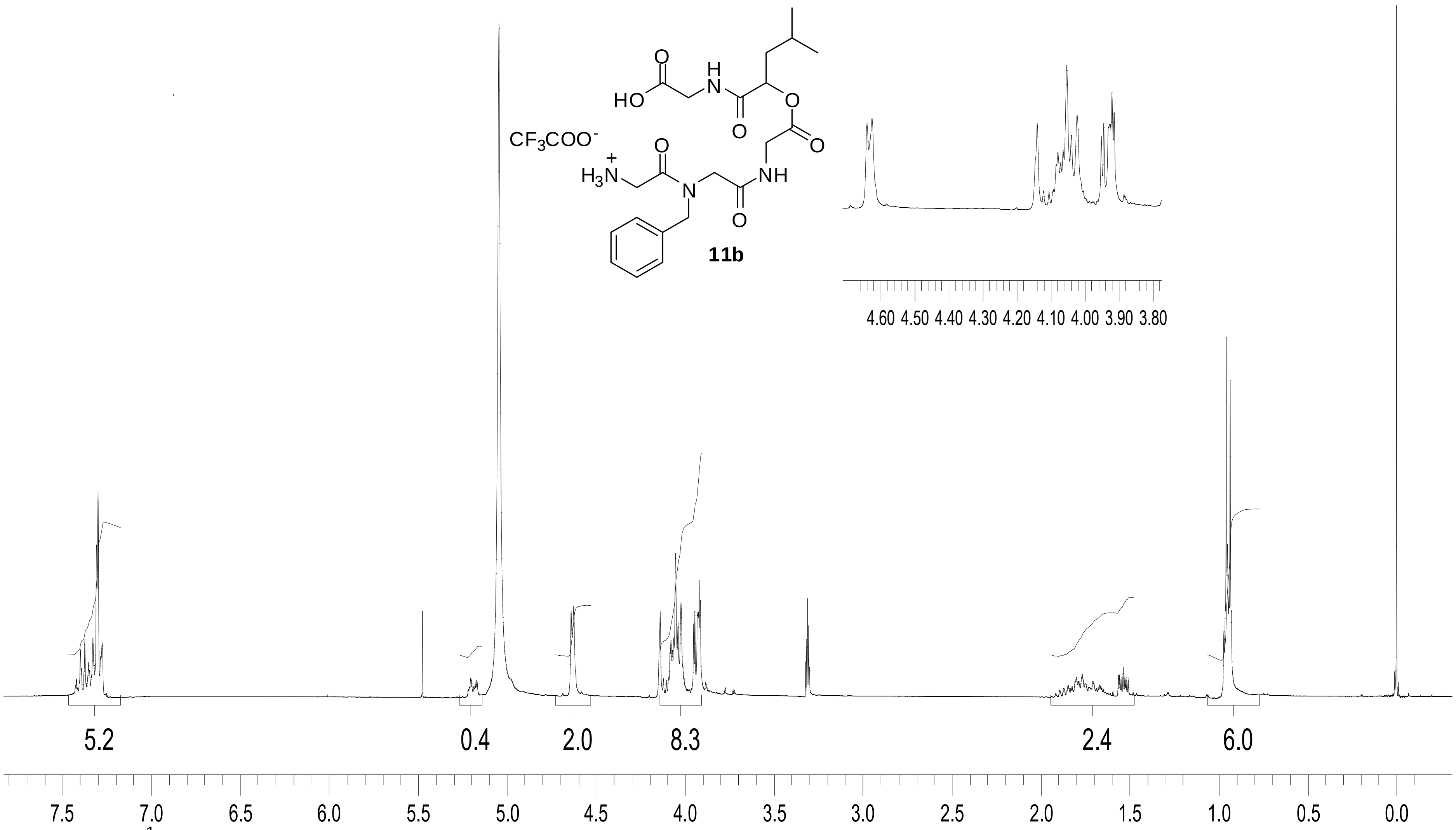

Figure S15: ^{1}H NMR (300 MHz, CD_3OD, presence of rotamers) spectrum of compound **11b**.

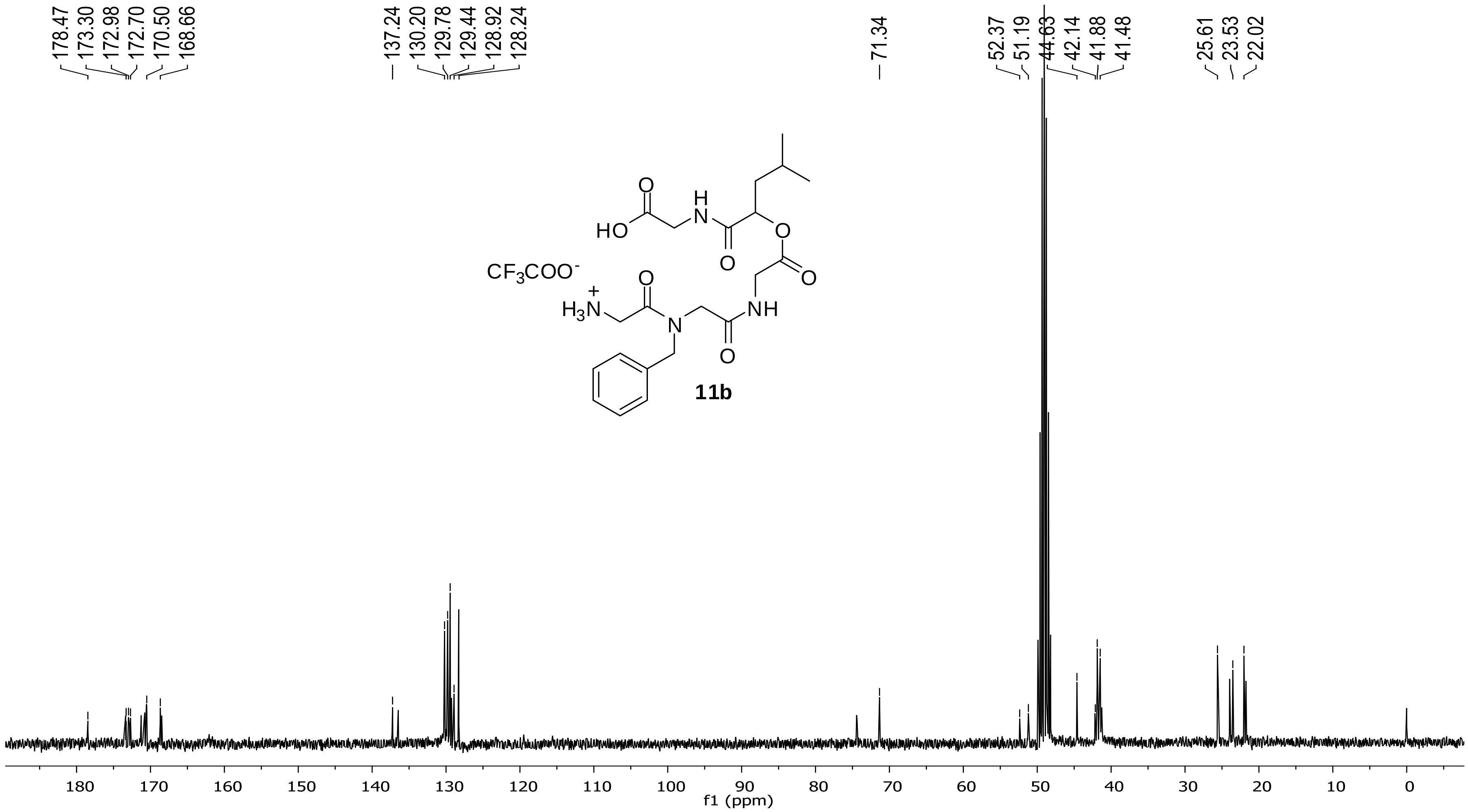

Figure S16: ^{13}C NMR (75.46 MHz, CD_3OD, presence of rotamers) spectrum of compound **11b**.

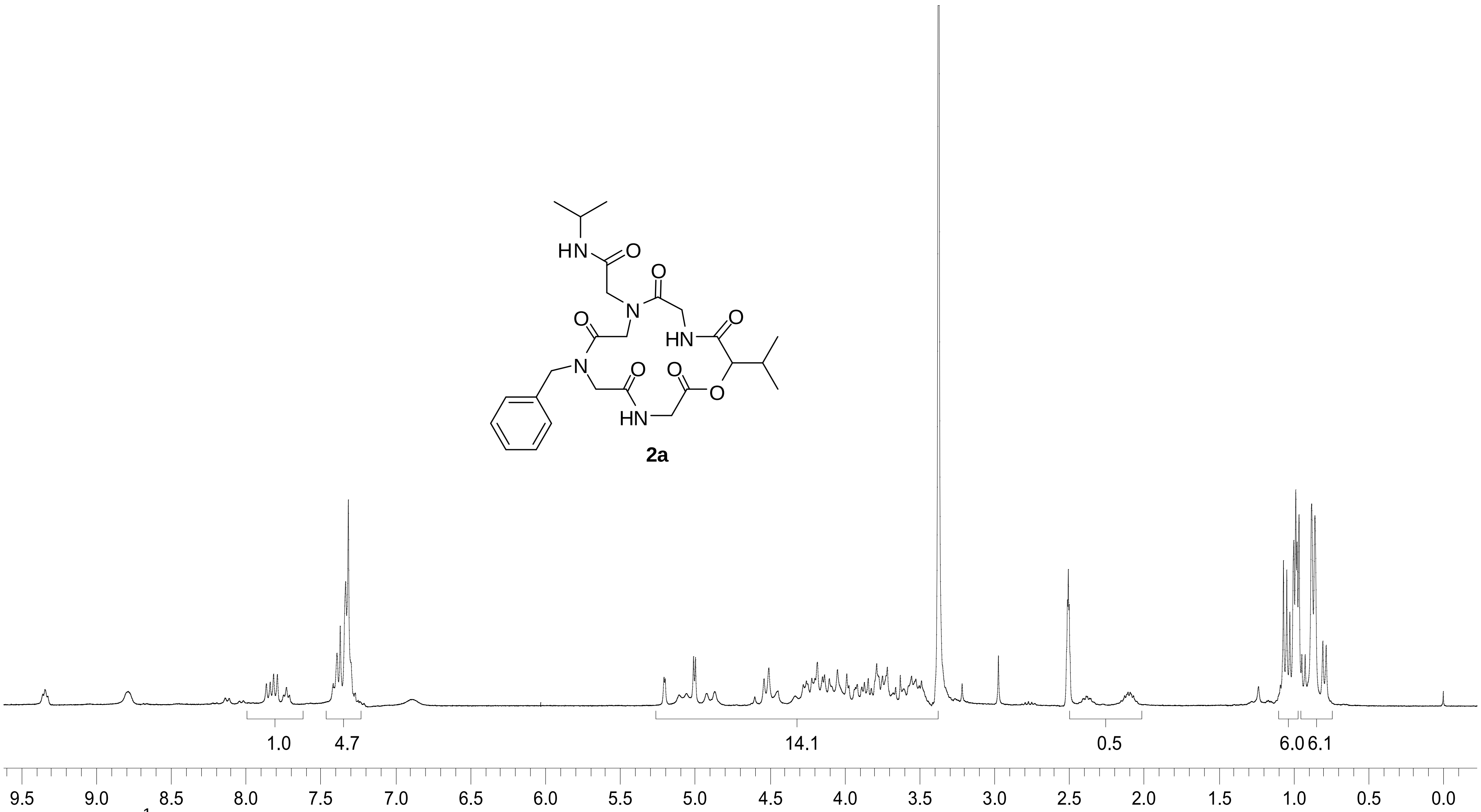

Figure S17: ^{1}H NMR (300 MHz, DMSO-d_6, presence of rotamers) spectrum of compound **2a**.

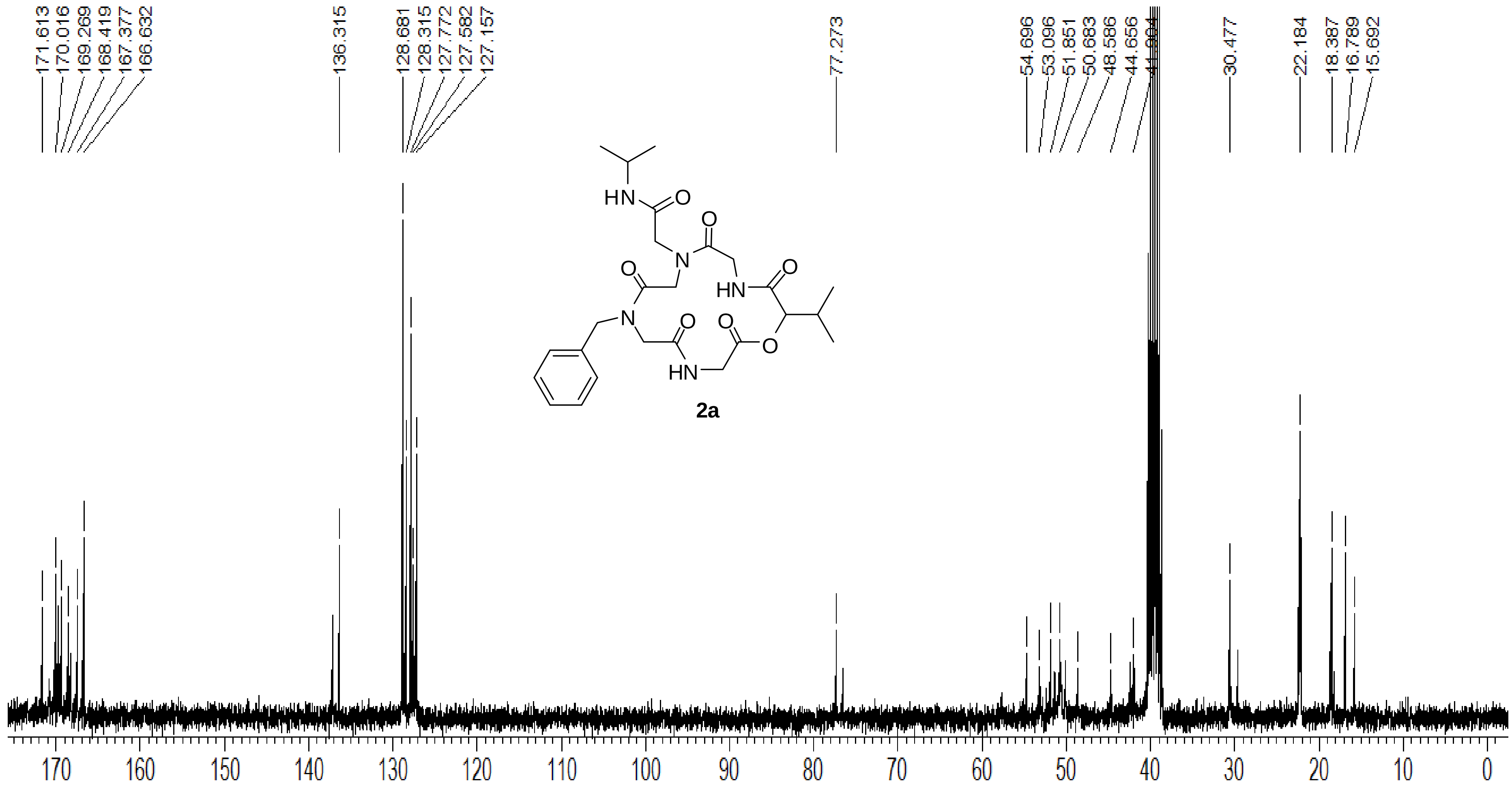

Figure S18: ^{13}C NMR (75.46 MHz, DMSO-d_6, presence of rotamers) spectrum of compound **2a**.

Equipment: MicroTOF **Manufacturer: Bruker Daltonics**

HV = 4500 V; Temp: 180 ºC; Flow: 240 μl/h

ESI$^+$

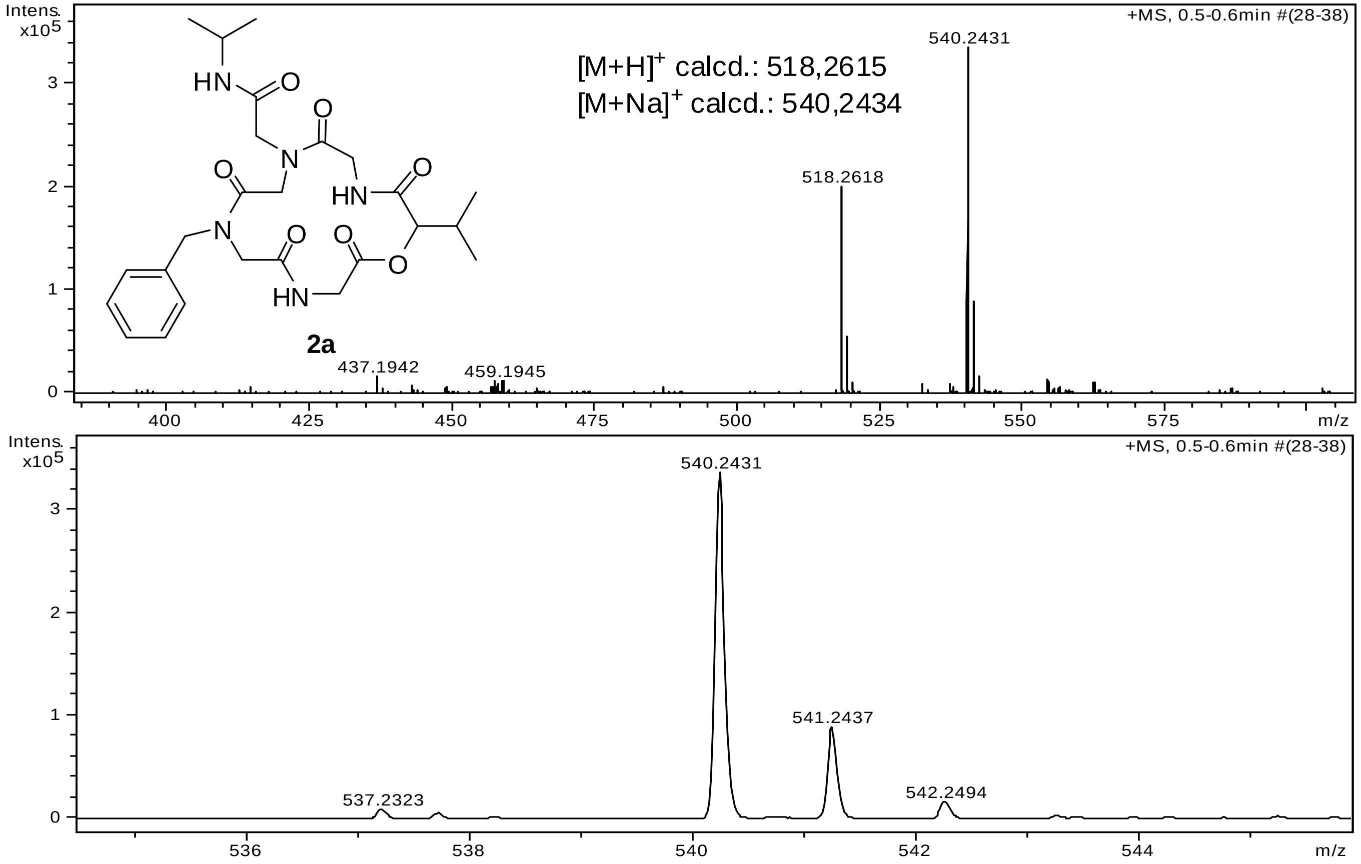

Figure S19: ESI-HRMS of compound **2a**.

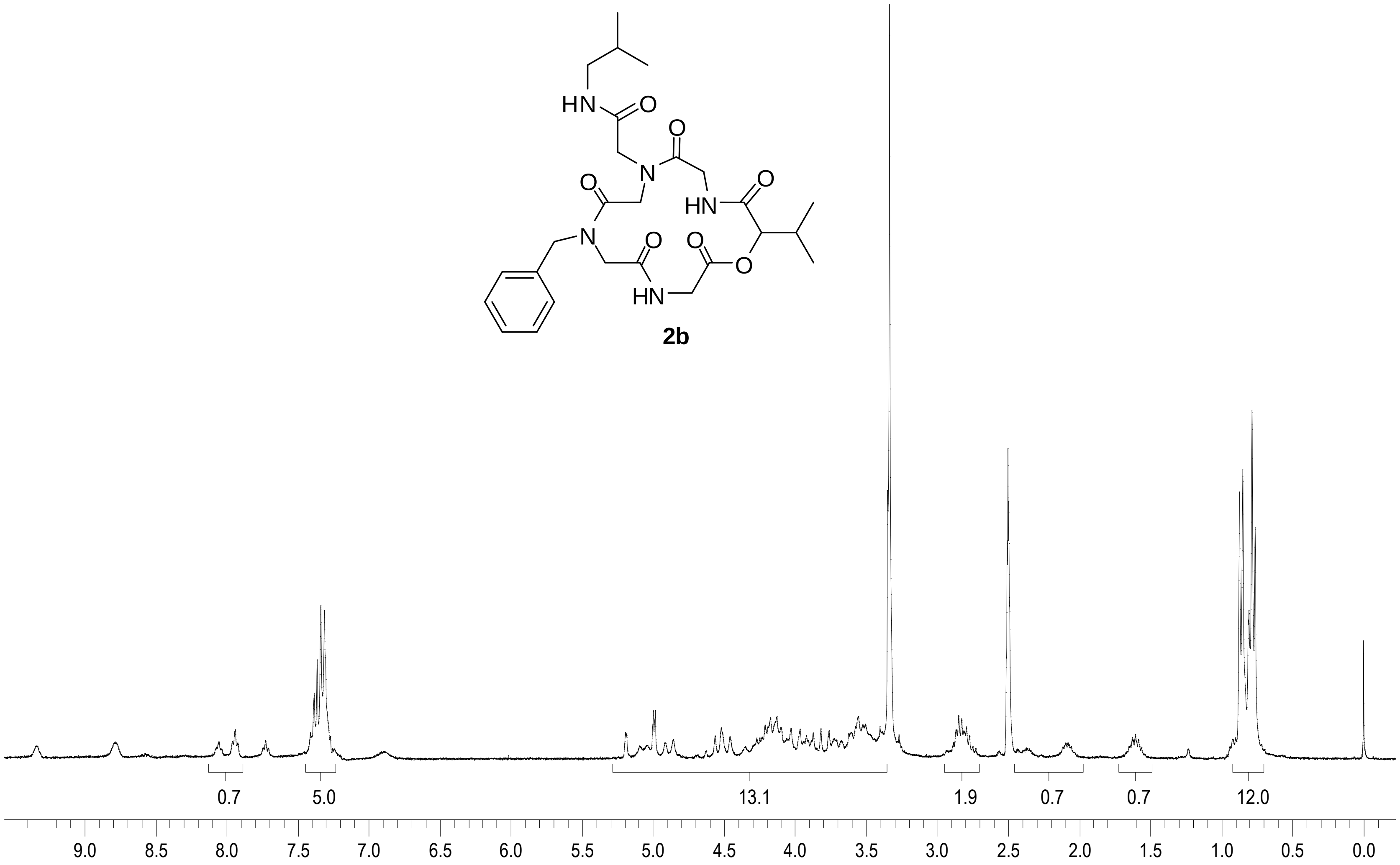

Figure S20: ^{1}H NMR (300 MHz, DMSO-d_6, presence of rotamers) spectrum of compound **2b**.

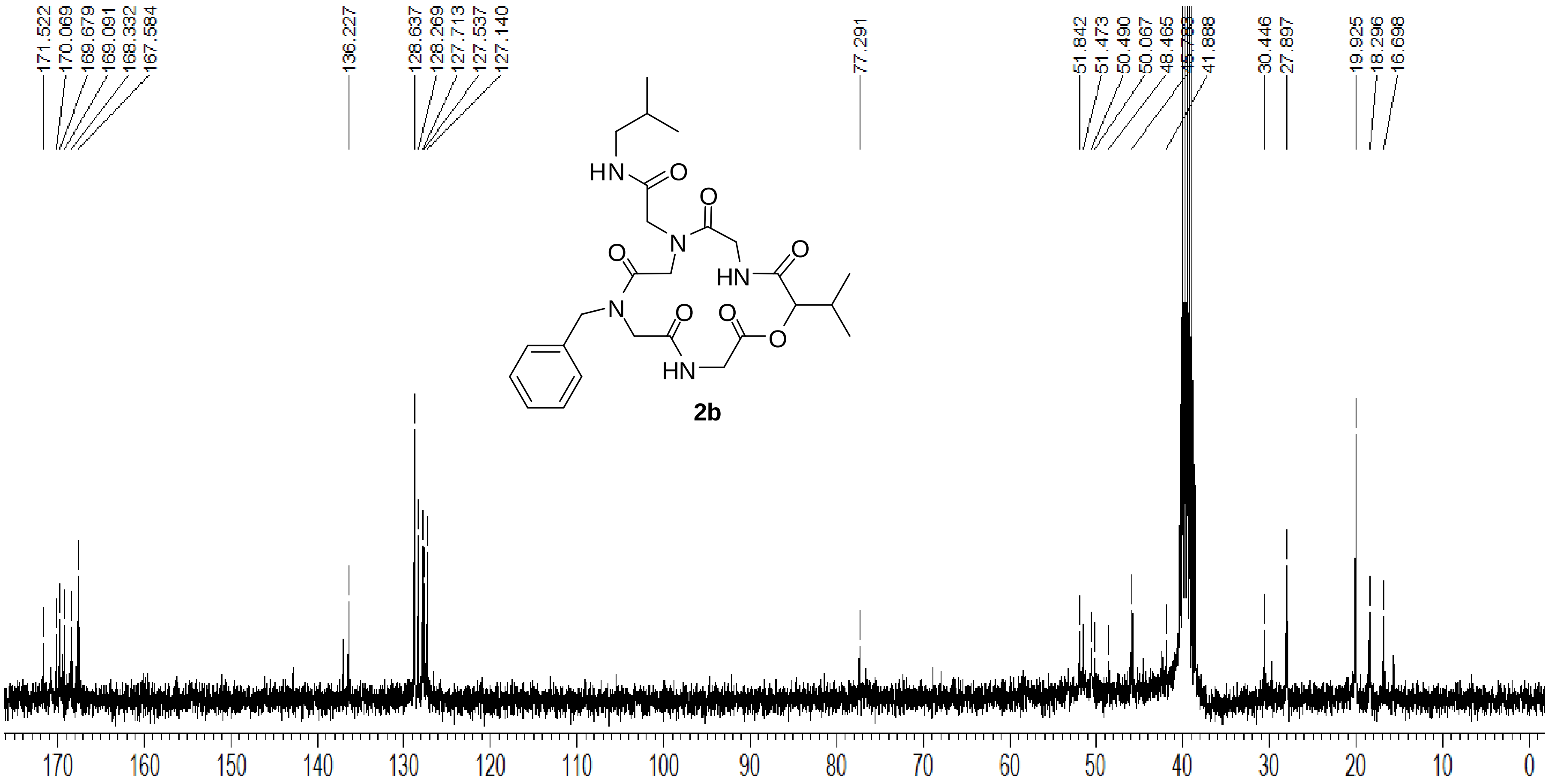

Figure S21: ^{13}C NMR (75.46 MHz, DMSO-d_6, presence of rotamers) spectrum of compound **2b**.

Equipment: MicroTOF **Manufacturer: Bruker Daltonics**

HV = 4500 V; Temp: 180 ºC; Flow: 180 μl/h

ESI^+ MeOH

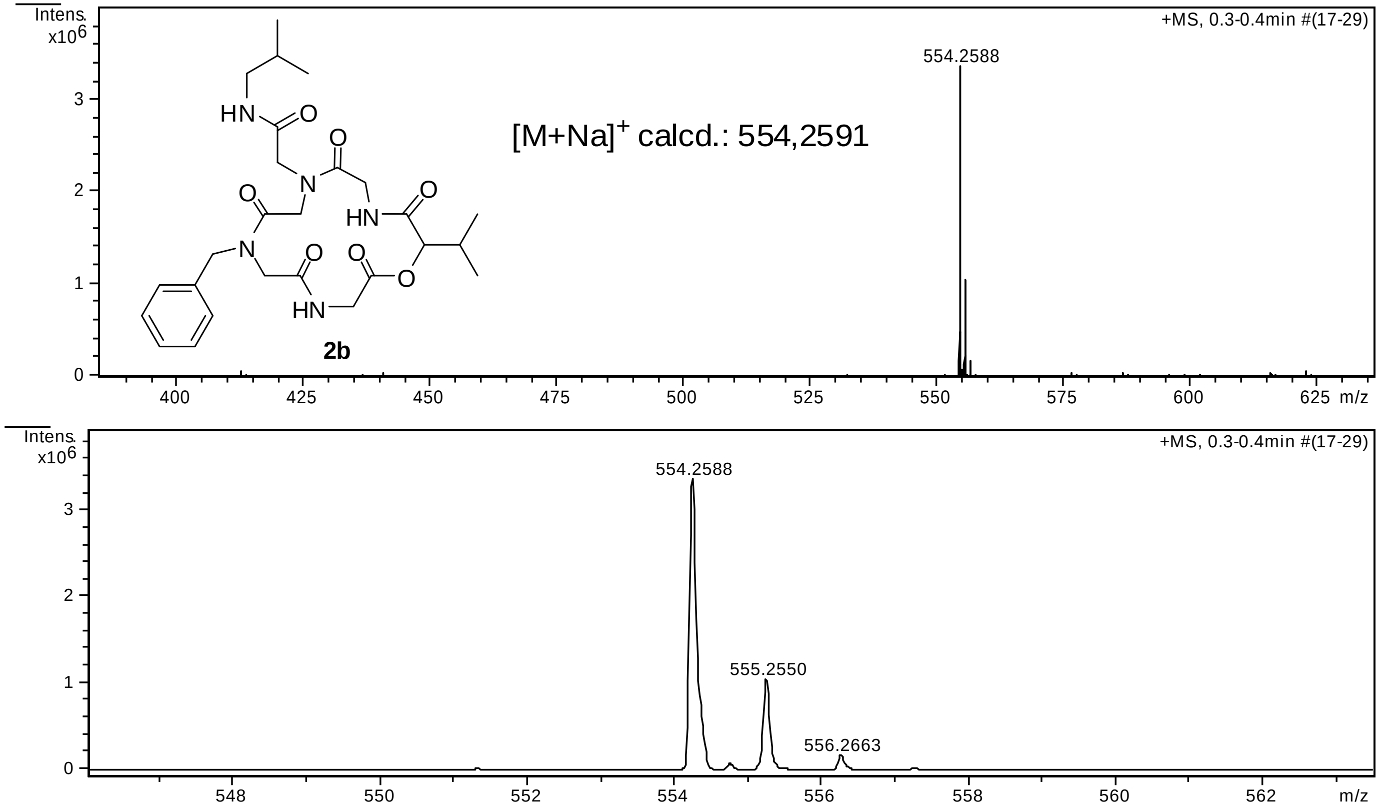

Figure S22: ESI-HRMS of compound **2b**.

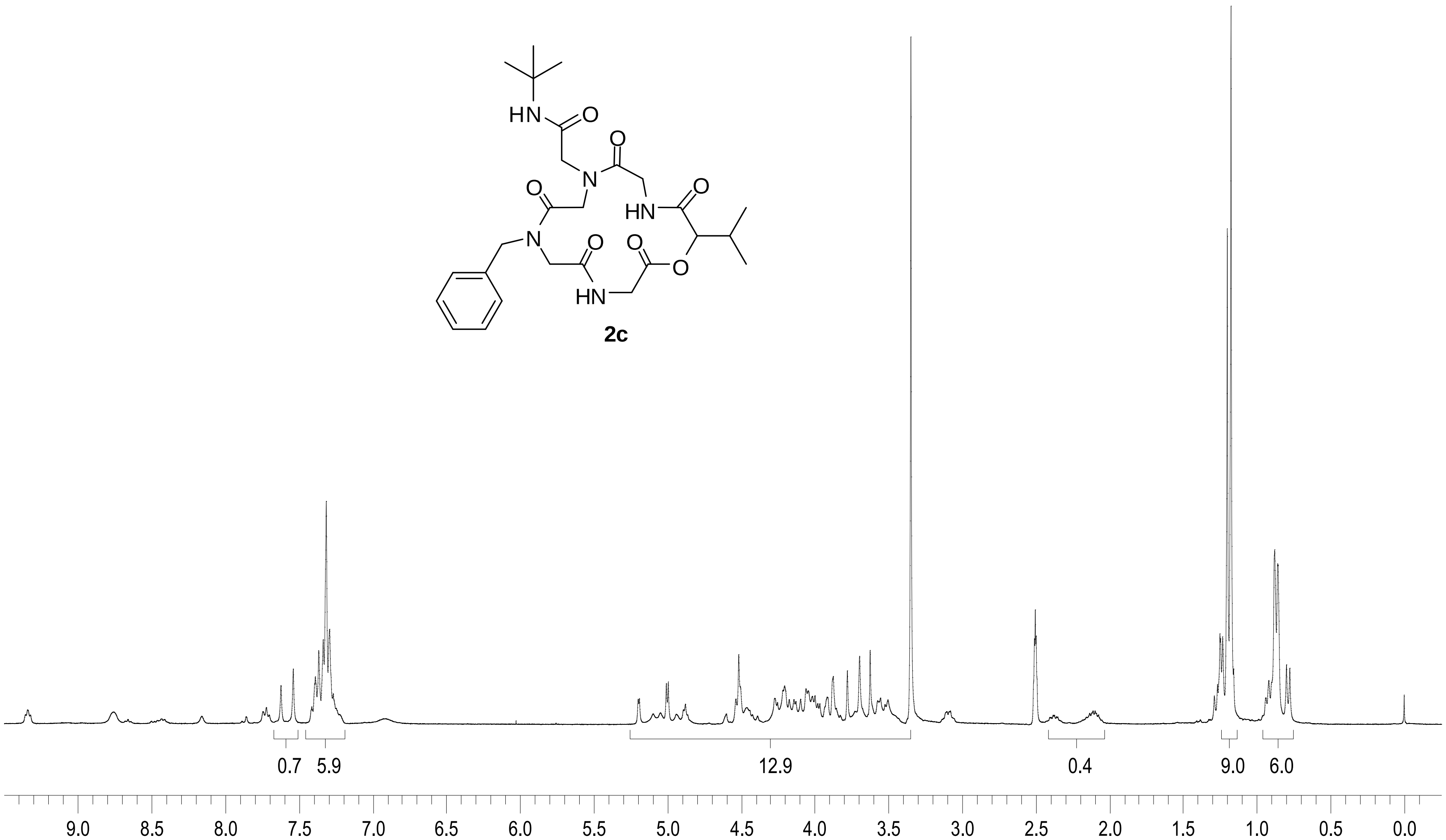

Figure S23: ^{1}H NMR (300 MHz, DMSO-d_6, presence of rotamers) spectrum of compound **2c**.

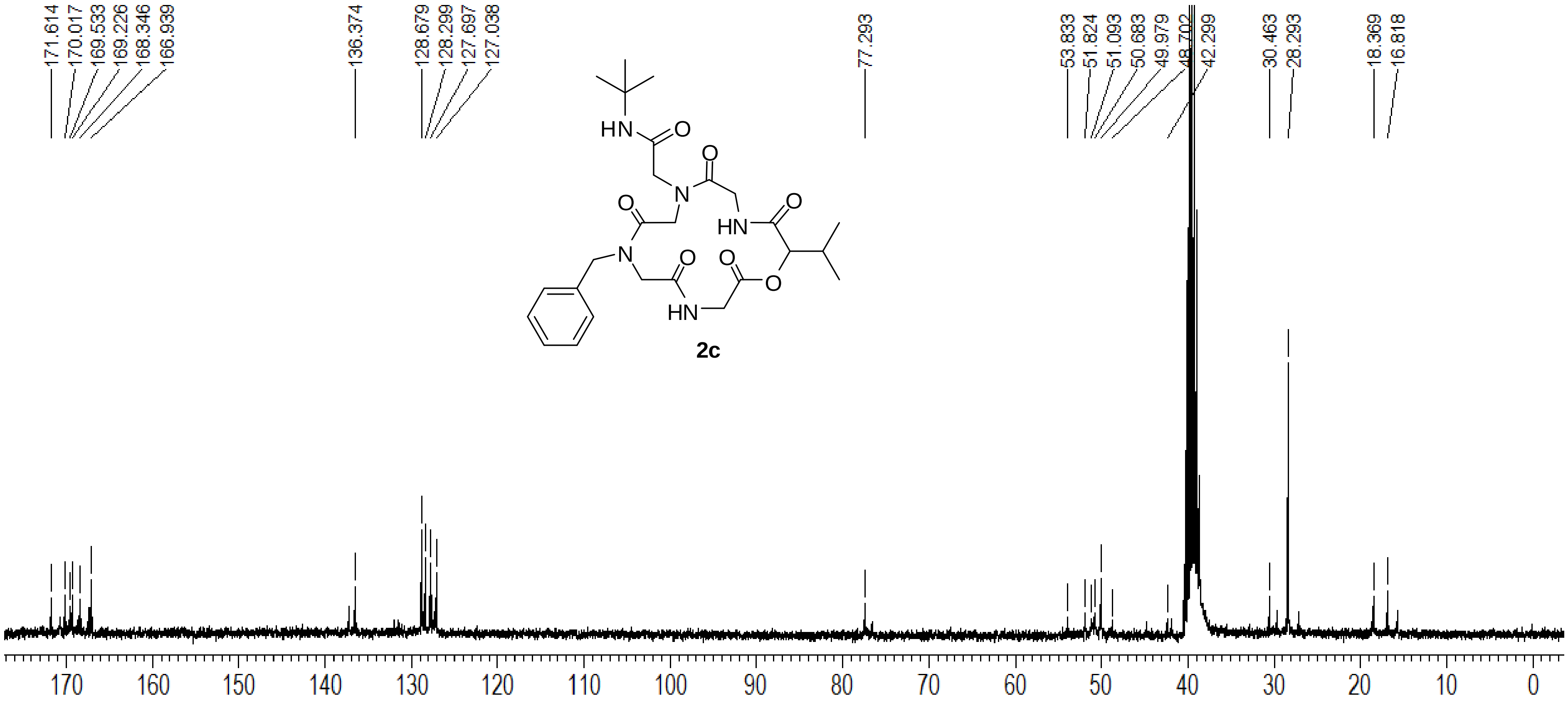

Figure S24: ^{13}C NMR (75.46 MHz, DMSO-d_6, presence of rotamers) spectrum of compound **2c**.

Equipment: MicroTOF **Manufacturer: Bruker Daltonics**

HV = 4500 V; Temp: 180 ºC; Flow: 240 μl/h

ESI^+ MeOH:H_2O 1:1

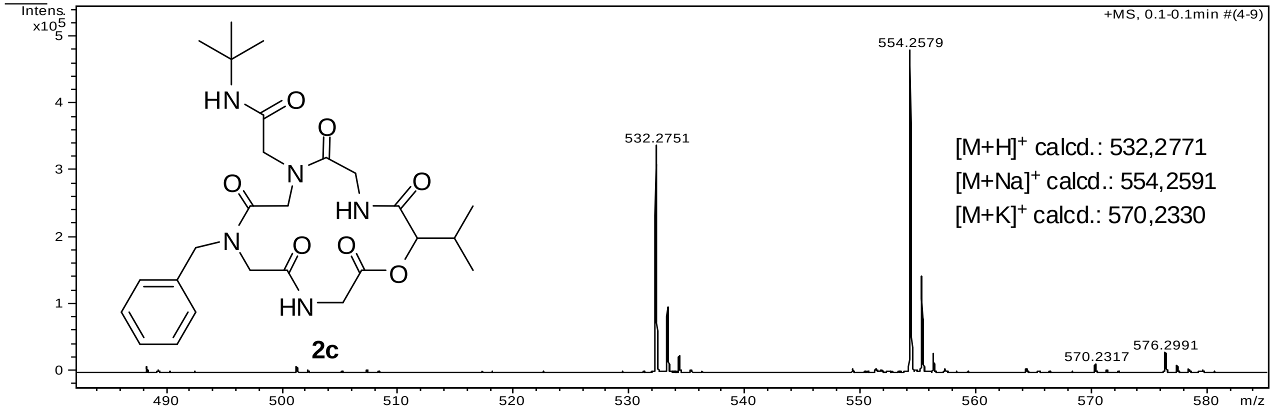

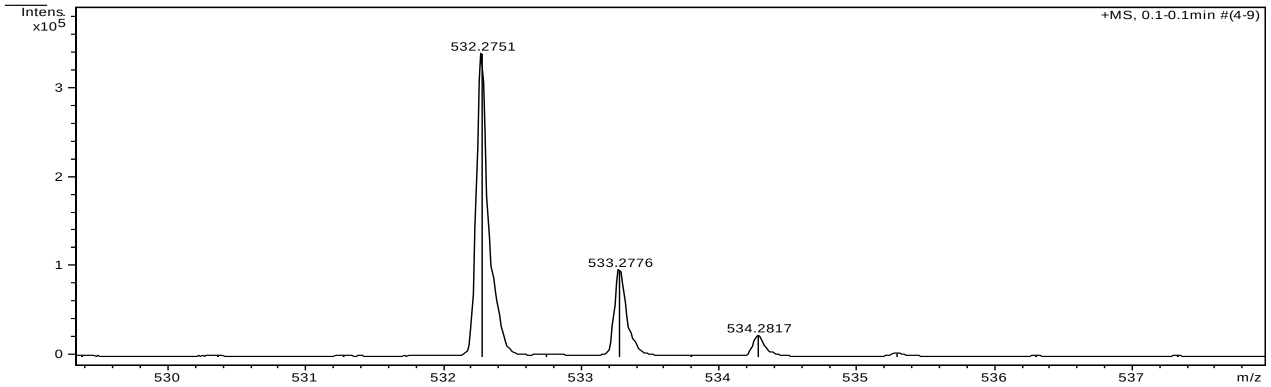

Figure S25: ESI-HRMS of compound **2c**.

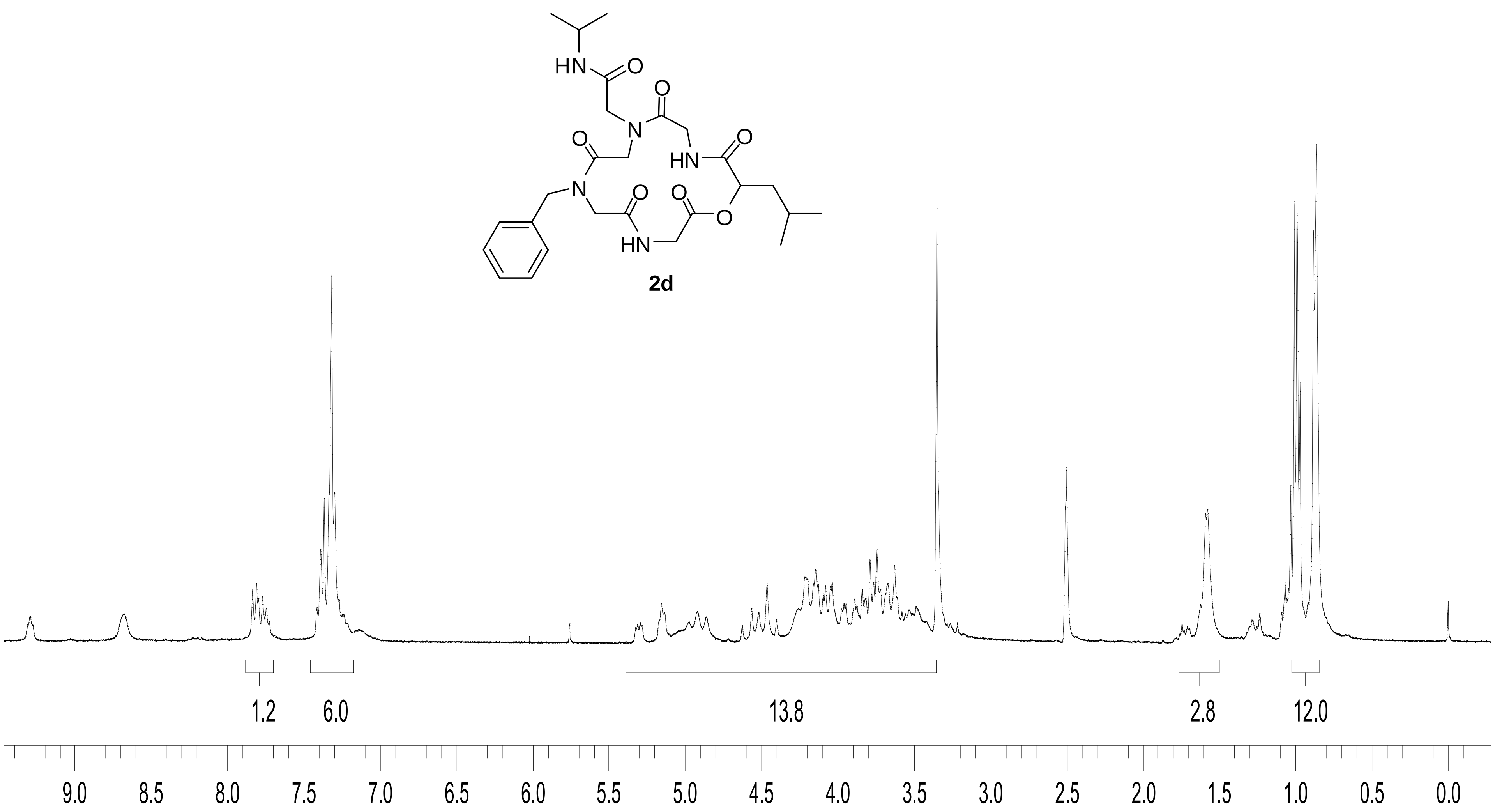

Figure S26: ^{1}H NMR (300 MHz, DMSO-d_6, presence of rotamers) spectrum of compound **2d**.

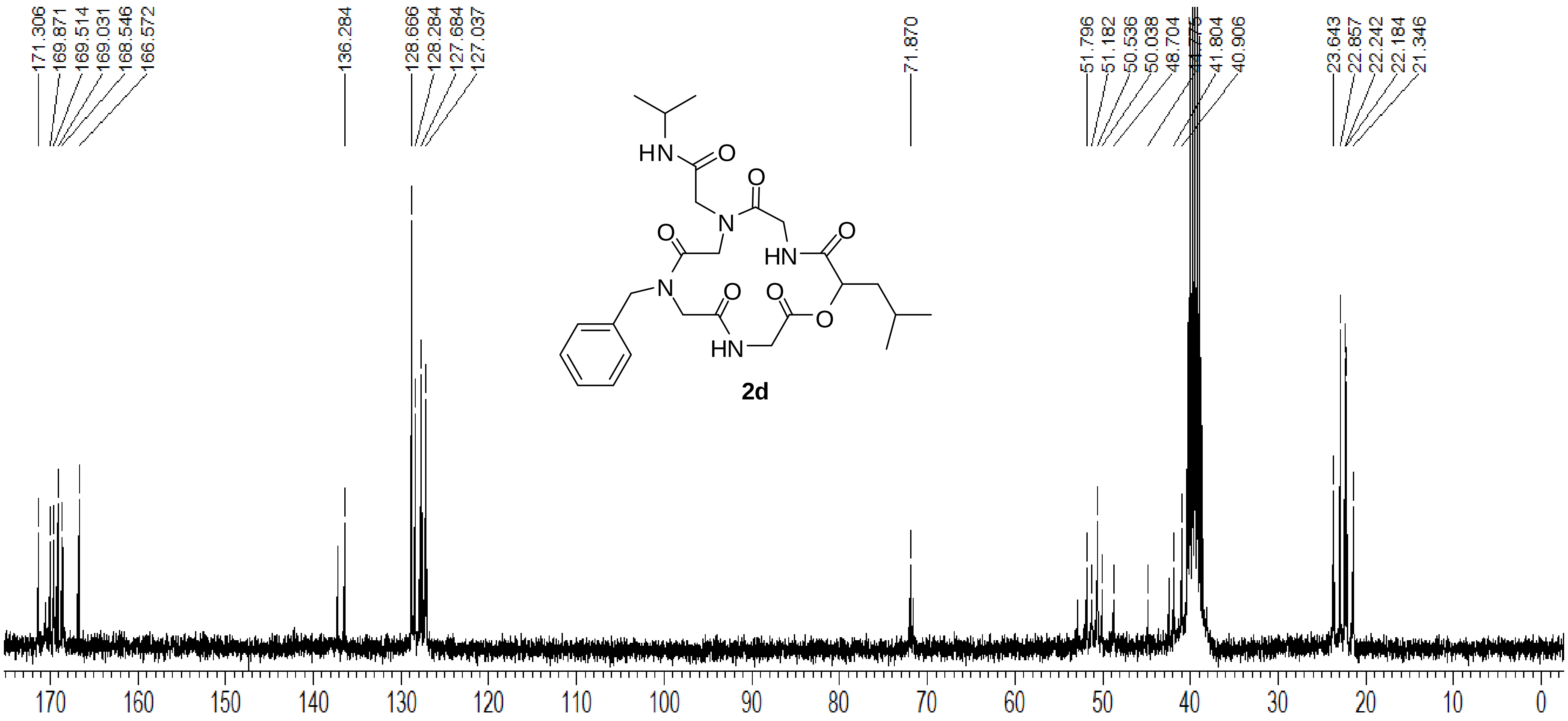

Figure S27: ^{13}C NMR (75.46 MHz, DMSO-d_6, presence of rotamers) spectrum of compound **2d**.

Equipment: MicroTOF **Manufacturer: Bruker Daltonics**

HV = 4500 V; Temp: 180ºC; Flow: 240 μl/h

ESI^+

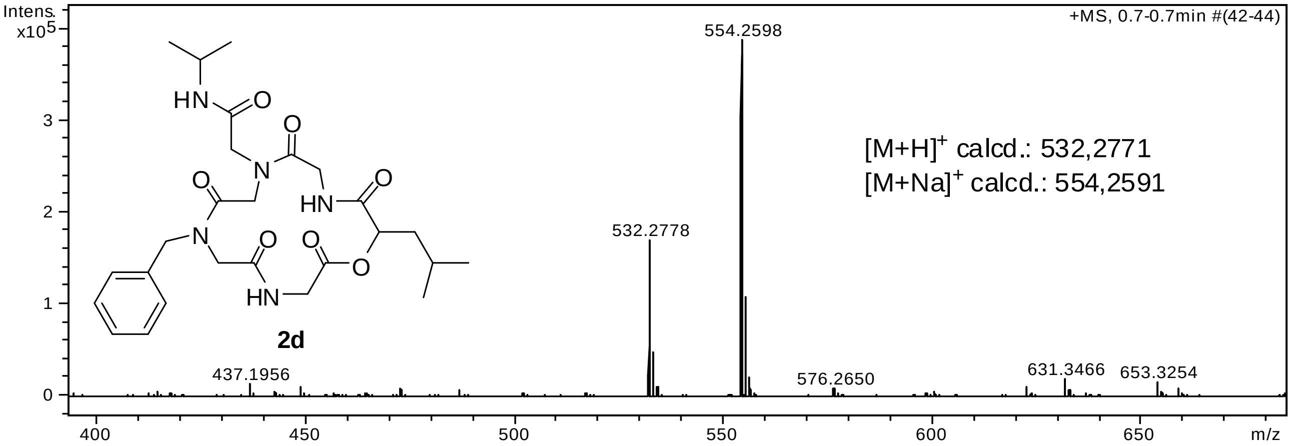

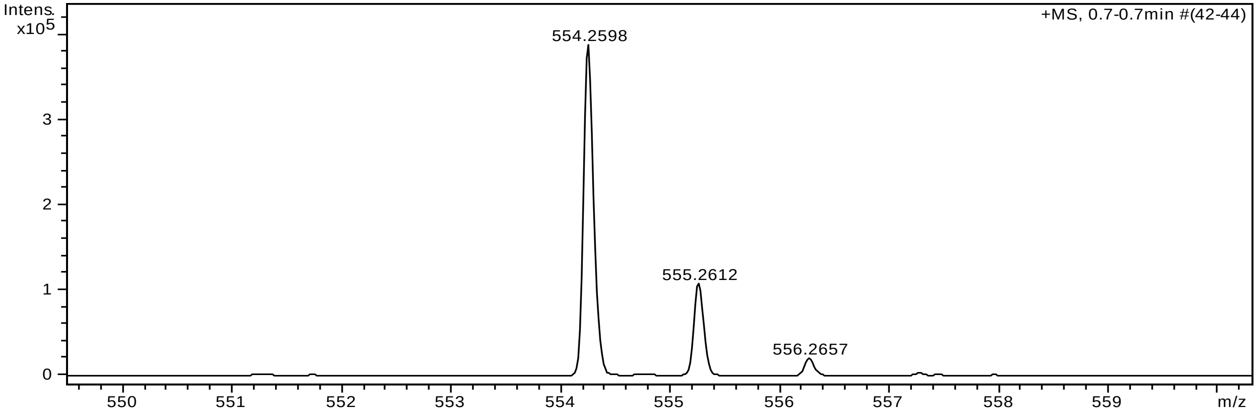

Figure S28: ESI-HRMS of compound **2d**.

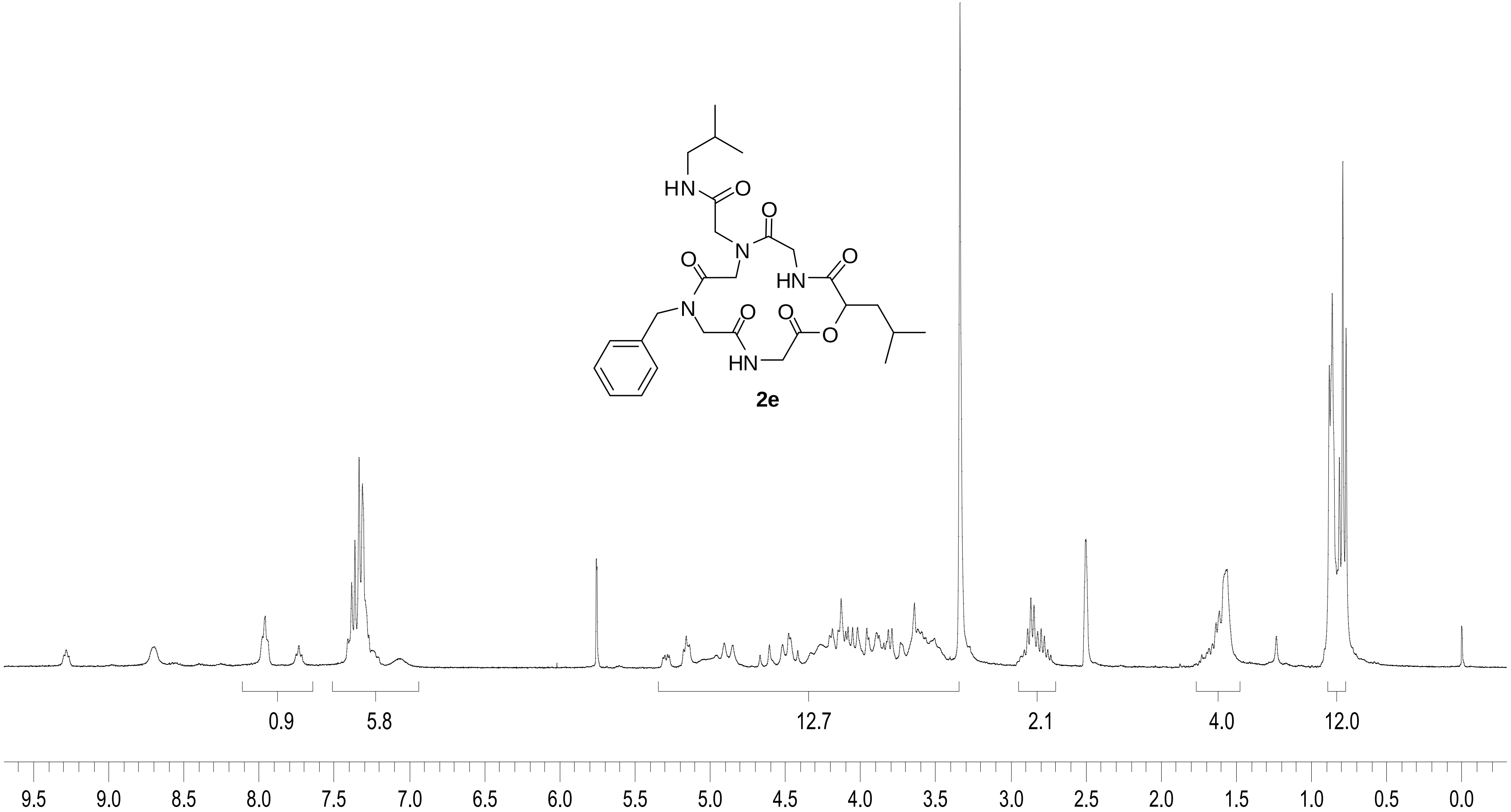

Figure S29: ^{1}H NMR (300 MHz DMSO-d_6, presence of rotamers) spectrum of compound **2e.**

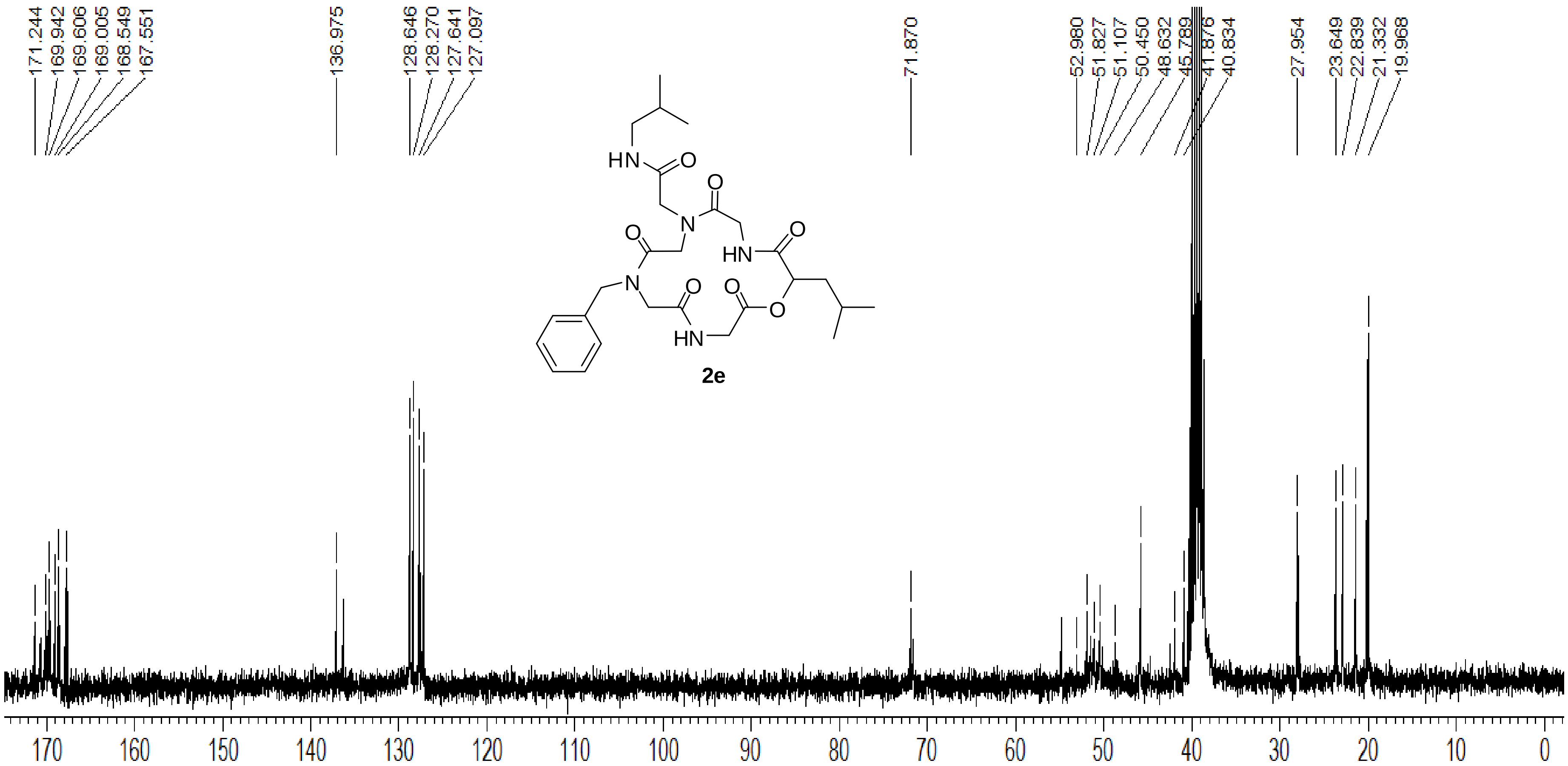

Figure S30: ^{13}C NMR (75.46 MHz, DMSO-d_6, presence of rotamers) spectrum of compound **2e**.

Equipment: MicroTOF **Manufacturer: Bruker Daltonics**

HV = 4500 V; Temp: 180 ºC; Flow: 240 μl/h

ESI^+ ACN 0.1% Formic acid

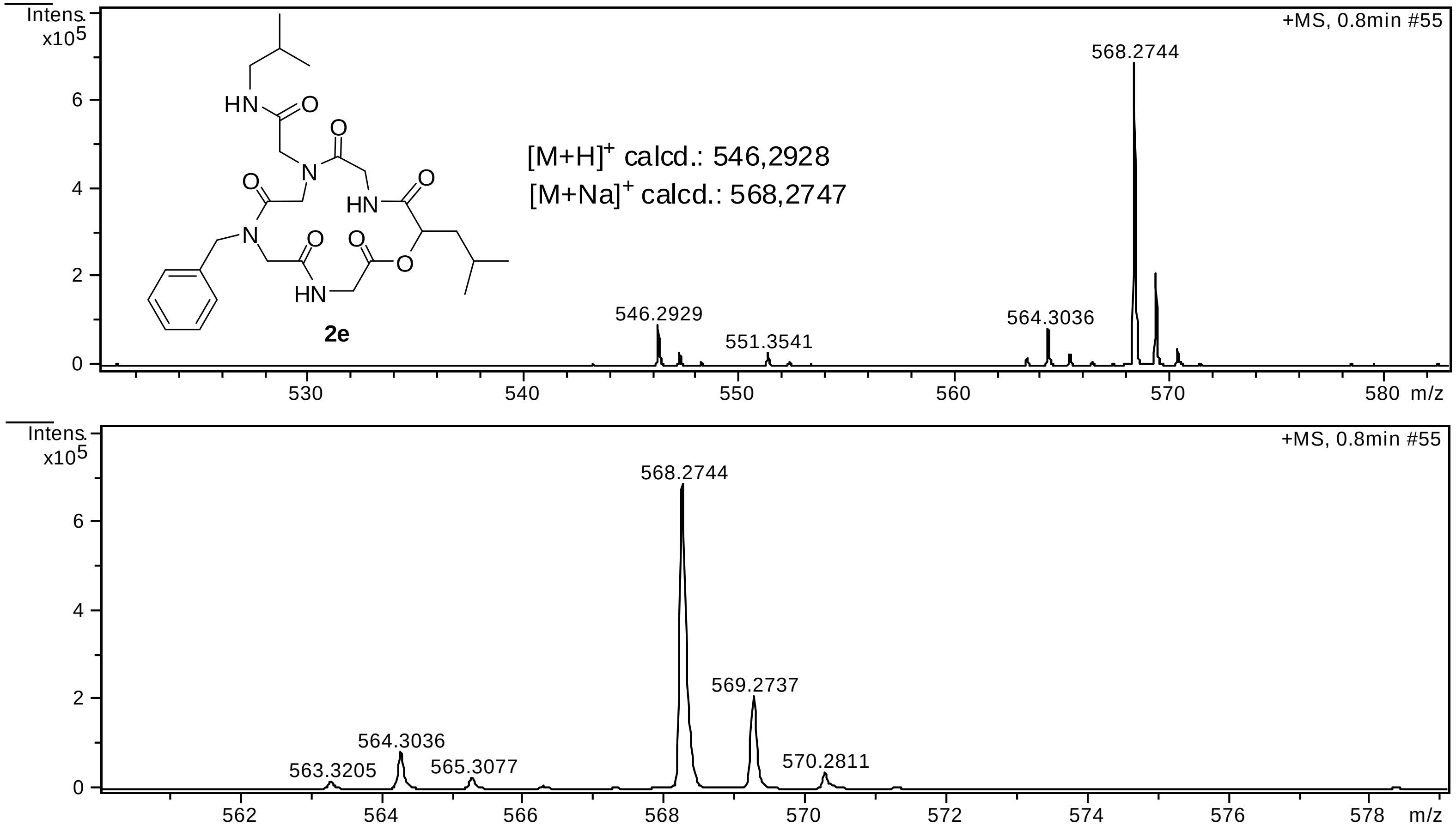

Figure S31: ESI-HRMS of compound **2e**.

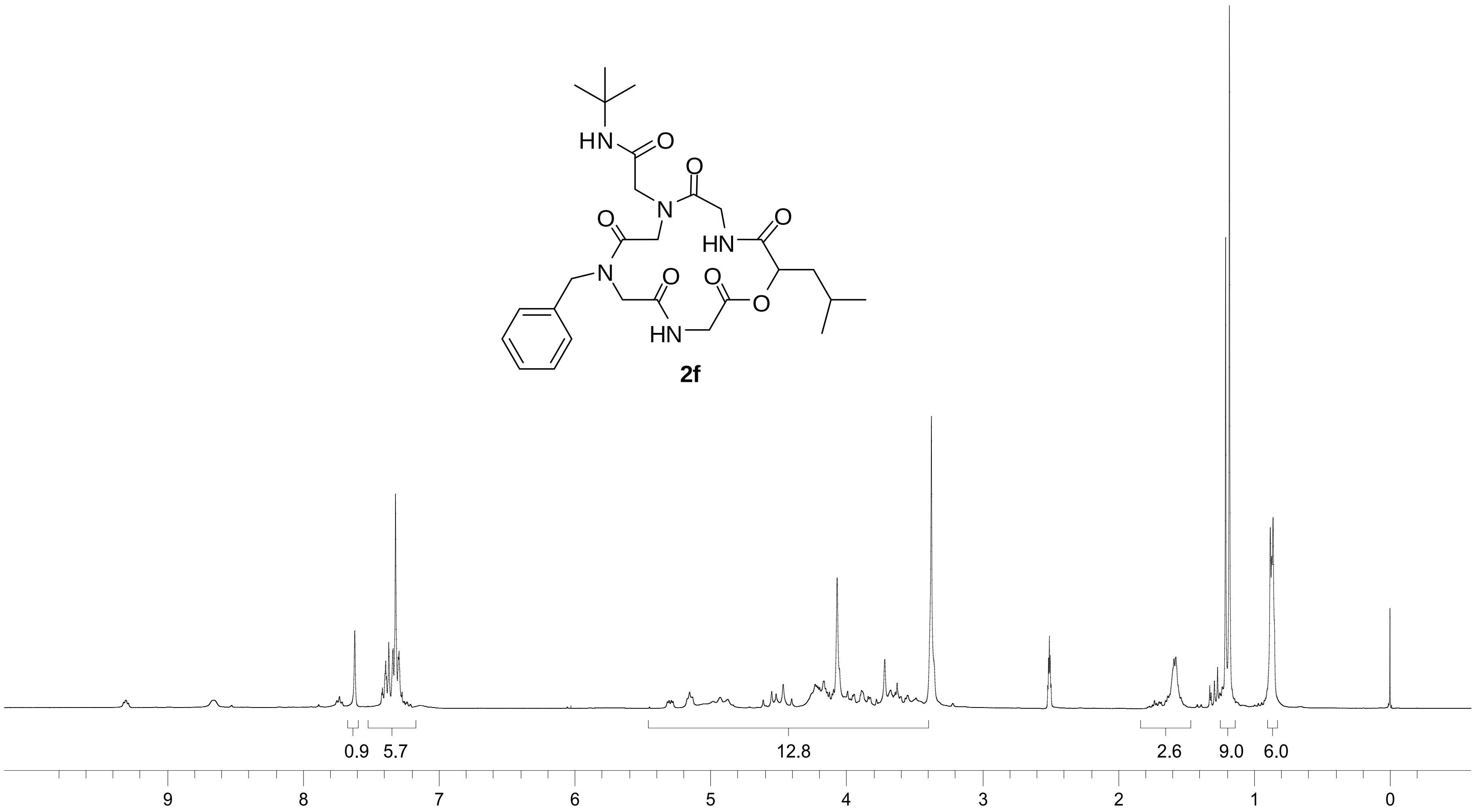

Figure S32: ^{13}C NMR (75.46 MHz, DMSO-d_6, presence of rotamers) spectrum of compound **2f**.

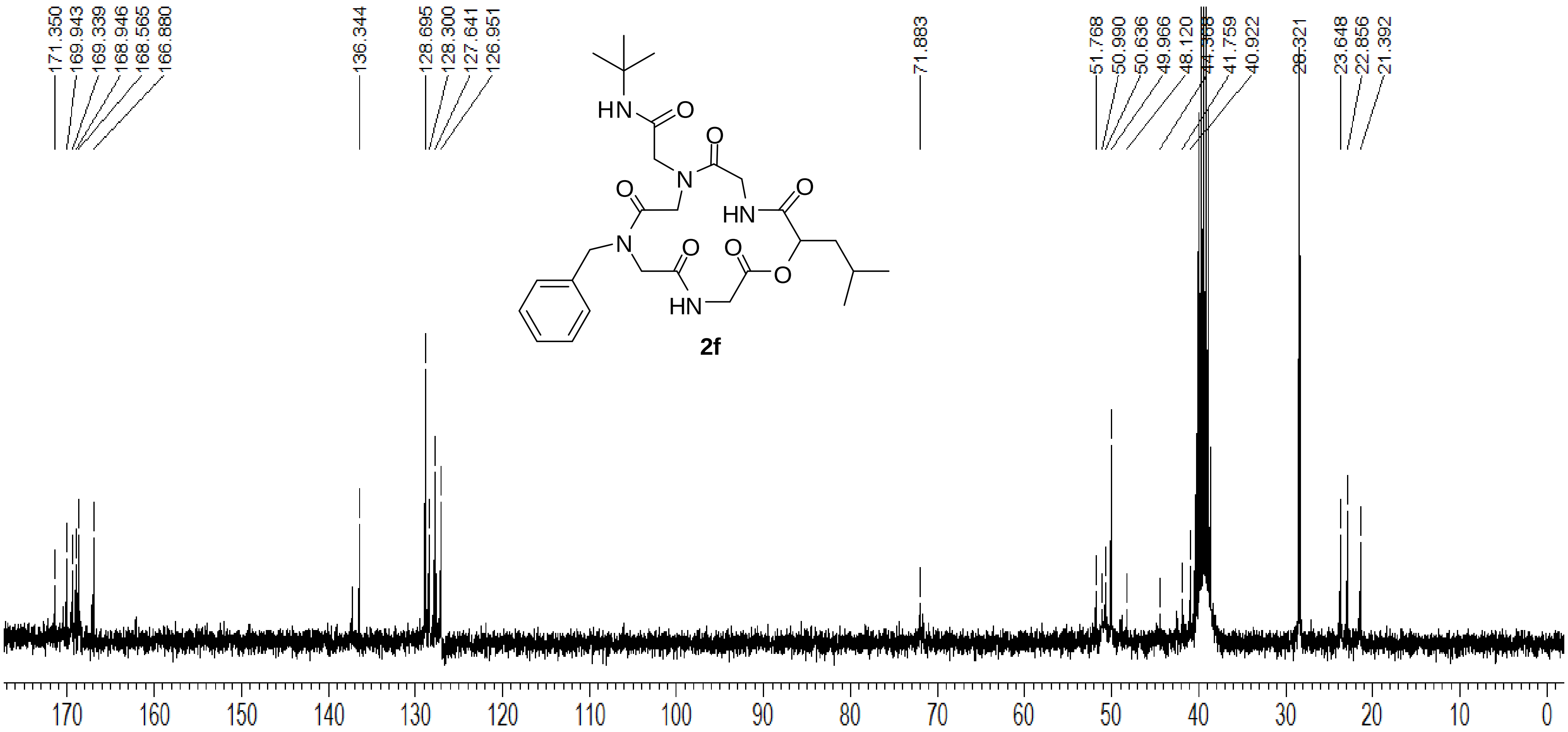

Figure S33: ^{13}C NMR (75.46 MHz, DMSO-d_6, presence of rotamers) spectrum of compound **2f**.

Equipment: MicroTOF **Manufacturer: Bruker Daltonics**

HV = 4500 V; Temp: 180 ºC; Flow: 240 μl/h

ESI$^+$ ACN 0.1% Formic acid

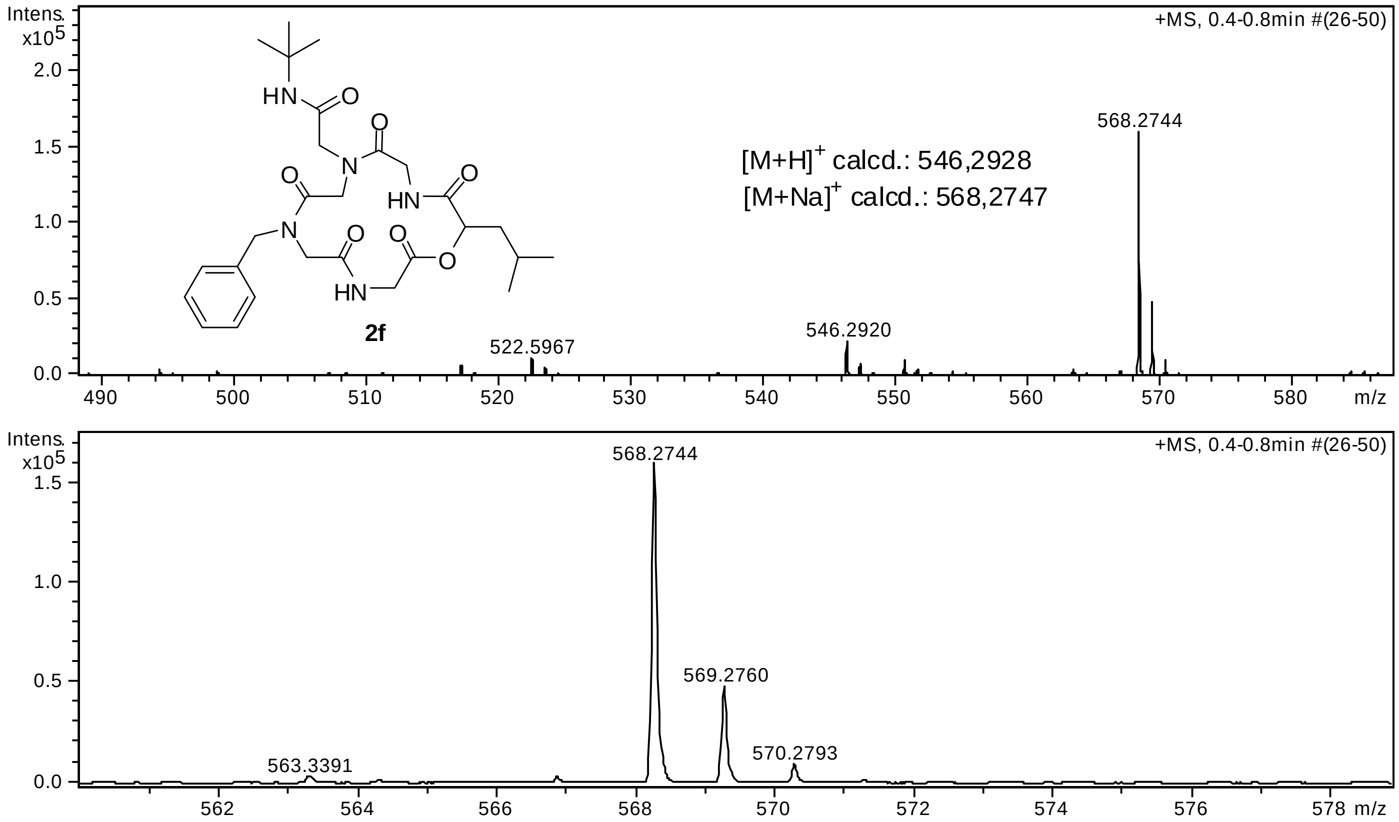

Figure S34: ESI-HRMS of compound **2f**.
